# Systematic Position Mapping of Split CRISPR‐Cas12a Activators Enables Highly Sensitive miRNA Detection and Cancer Cell Stratification

**DOI:** 10.1002/advs.76576

**Published:** 2026-07-23

**Authors:** Xiaoyan Tang, Zhe Li, Yuning Lu, Miao Ma, Zijian Mo, Jiajun Ke, Xinyu Luan, Tiangang Luan, Junqiu Zhai

**Affiliations:** ^1^ Key Laboratory of Chinese Medicinal Resource from Lingnan, Ministry of Education School of Pharmaceutical Sciences Guangzhou University of Chinese Medicine Guangzhou P. R. China; ^2^ School of Environmental and Chemical Engineering Wuyi University Jiangmen P. R. China; ^3^ State Key Laboratory of Biocontrol, School of Life Sciences Sun Yat‐sen University Guangzhou P. R. China; ^4^ Guangdong Provincial Key Laboratory of New Drug Design and Evaluation, School of Pharmaceutical Sciences Sun Yat‐Sen University Guangzhou P. R. China

**Keywords:** CRISPR/Cas12a, enzyme activity regulation, split activators

## Abstract

Amplification‐free Cas12a diagnostics with split crRNA enable rapid and programmable target recognition, yet insufficient understanding of DNA activator architecture prevents predictable control over *trans*‐cleavage activity and sensitivity. Here we systematically map over 200 split DNA activator configurations by introducing nicks at every position across both strands. The mapping reveals that target strand nicks suppress activity with position‐dependent severity, while non‐target strand nicks enhance activity. Guided by these rules, we engineer an optimized split activator pair that achieves attomolar microRNA detection (LOD: 112 aM), ∼480‐fold higher sensitivity than intact activators. The enhanced sensitivity supports multiplexed live‐cell profiling of five miRNAs for machine learning‐based cancer cell stratification, and is further generalized to non‐nucleic acid targets, including APE1 enzyme (0.0073 U/L) and HClO (2.37 pM) through position‐informed cleavable sites. This work provides a generalizable methodology for engineering CRISPR‐Cas12a performance across diagnostic and biosensing applications.

## 1 Introduction

Rapid and accurate molecular detection underpins modern diagnostics, yet conventional methods often require costly pre‐amplification steps that increase assay complexity and turnaround time [1, 2, 3]. The CRISPR‐Cas12a system offers a promising solution through its intrinsic signal amplification mechanism. Upon binding target dsDNA activators via crRNA‐guided base pairing, Cas12a initiates collateral cleavage of single‐stranded DNA reporters, generating amplified fluorescent signals without pre‐amplification [4, 5]. This unique *trans*‐cleavage activity has positioned CRISPR‐Cas12a as a powerful platform for sensitive nucleic acid detection in clinical diagnostics [6], environmental monitoring [7, 8], and food safety [9, 10]. However, realizing the full potential of this system requires achieving programmable control over *trans*‐cleavage efficiency to rationally tune signal output and maximize detection sensitivity across diverse applications.

Recent efforts to modulate Cas12a activity have pursued two complementary strategies. Topological engineering approaches, including bubble‐structured activators [11], three‐dimensional steric activators [7], and G‐quadruplex‐modified crRNAs [12], exploit conformational constraints to tune *trans*‐cleavage efficiency, though these modifications predominantly suppress rather than enhance activity and often exhibit slow kinetics [11, 13, 14, 15]. More recently, split DNA activators have been explored, yielding inconsistent effects on Cas12a activity depending on nick position [13, 16]. Parallel to these activator‐centric strategies, split crRNA architectures have emerged as a versatile strategy for enhancing specificity and enabling molecular logic. However, the interplay between split DNA activators and split crRNA assemblies introduces unique conformational dynamics that complicate activity prediction [17, 18]. Existing studies have focused on examining these elements in isolation or at selected positions, leaving the combinatorial position‐activity relationship unmapped [19, 20]. Without a systematic understanding of how nick positioning across both DNA strands controls *trans*‐cleavage efficiency within split crRNA systems, predictable engineering of signal output remains elusive.

Here, we address this gap through systematic mapping of split DNA activators. By introducing nicks at every position across the target strand (TS) and non‐target strand (NTS), we construct a complete regulatory atlas spanning over 200 distinct configurations. Three key findings emerge. First, we establish quantitative design rules: TS nicks universally suppress activity with position‐dependent severity, while NTS nicks enhance activity when combined with split TS. Second, kinetic dissection reveals that nick positioning controls both RNP binding and *cis*‐cleavage efficiency, which collectively gate *trans*‐cleavage output. Third, applying these principles, we engineer split activators achieving attomolar microRNA detection (LOD: 112 aM), ∼480‐fold improvement over intact activators. Beyond sensitivity enhancement, we demonstrate cancer cell stratification via multiplexed miRNA profiling and extend the framework to non‐nucleic acid targets through position‐informed cleavable sites, establishing generalizable principles for engineering CRISPR‐Cas12a biosensors.

## 2 Results

### 2.1 Position‐Dependent Nick Sites Modulate Cas12a *Trans*‐Cleavage by Split dsDNA Activators

**FIGURE 1:**
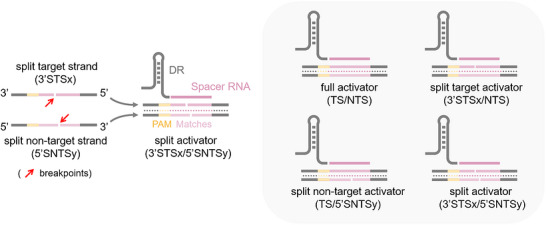
Engineering split activator strategies for probing Cas12a function. Schematic representation of nicks on dsDNA activators (yellow = PAM region, pink = match region). Gray area represents all possible combinations of nick site presence and absence in TS and NTS.

To establish design principles for optimizing split dsDNA activators, we conducted a comprehensive systematic investigation of nick site effects across the entire dsDNA activator sequence. We introduced nicks at every position in both the target strand (TS) and the non‐target strand (NTS) of the dsDNA activator to map the complete position‐activity landscape (Figure 1). As illustrated in Figure S1, the target strand was split into two fragments by sequentially shifting the nick position x one base at a time (3'STSx, where x indicates the nick site located immediately after the x th base from the 3' end of the recognition domain). Similarly, the non‐target strand was divided at position y (5'SNTSy). This comprehensive matrix design enabled us to test all possible nick site combinations and identify synergistic effects between TS and NTS nicks.

**FIGURE 2:**
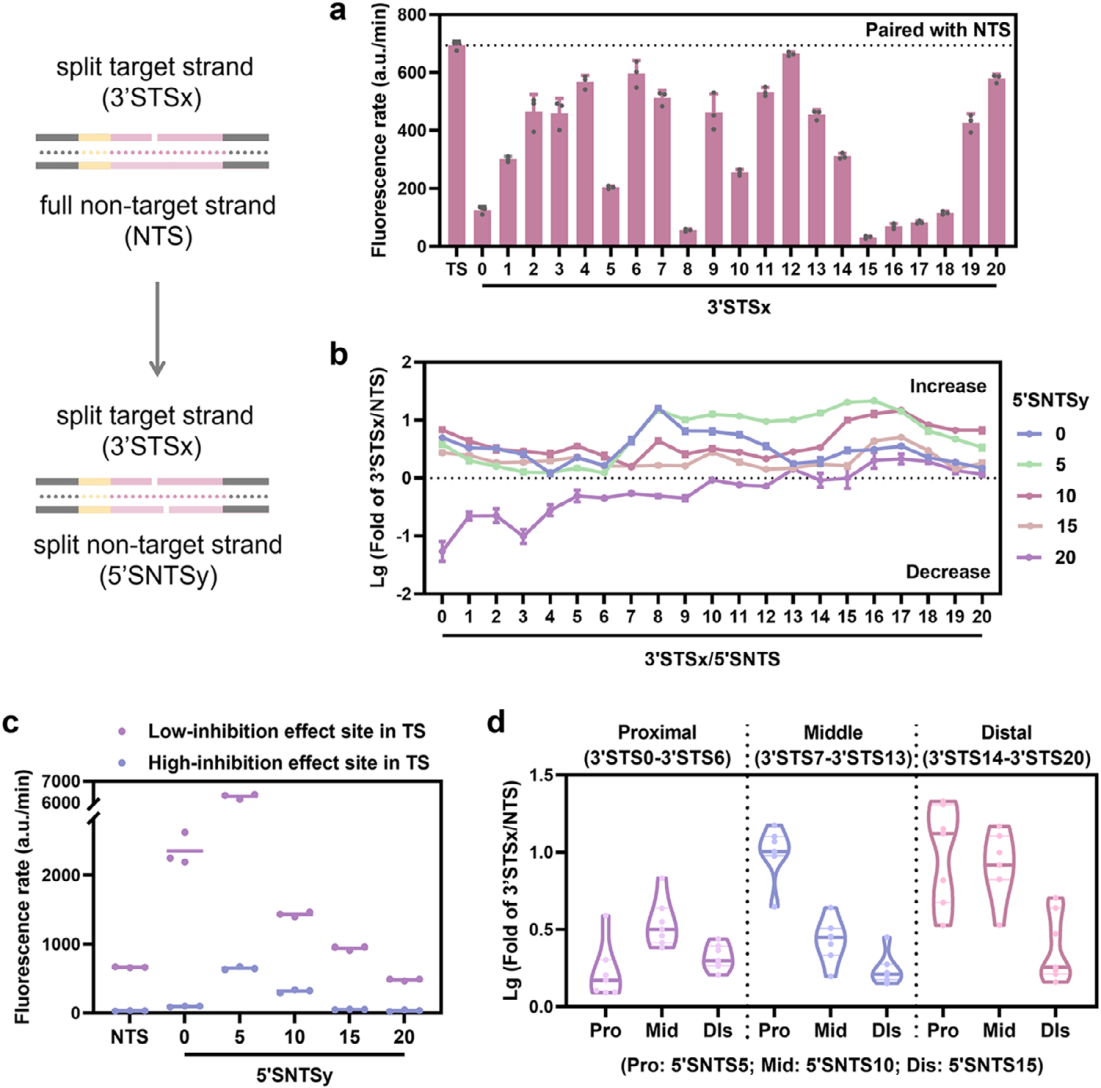
The inhibitory effect of nicked target strand on *trans*‐cleavage activity is alleviated by broken non‐target strand. (a)*Trans*‐cleavage activity of Cas12a with 3’STSx/NTS. (b) Fold change in activity of 3’STSx/5’SNTSy compared to 3’STSx/NTS. The dashed line denotes the baseline, with positions above and below representing activation and suppression, respectively. (c) Cas12a activity change under 3’STS12/5’SNTSy (high promotive effect) and 3’STS15/5’SNTSy (low promotive effect). (d) Synergistic regulation by TS and NTS nick positions in different regions relative to PAM. Data represent mean ± SD of three technical replicates; reactions were performed at 37°C for 120 min.

By combining 3’STSx with NTS, 5’SNTS0, 5’SNTS5, 5’SNTS10, 5’SNTS15, and 5’SNTS20 to form split dsDNA activators, we first assessed the impact of 3’STSx on Cas12a *trans*‐cleavage activity in a split crRNA system (Figure S2). When the TS was nicked but paired with a full‐length NTS, Cas12a *trans*‐cleavage activity was generally suppressed, as reflected by reduced fluorescence signals (Figure 2a). This inhibition likely arises from decreased stability of spacer RNA binding, which delays R‐loop assembly and impedes Cas12a activation. In contrast, when TS nicks were combined with split NTSs, the inhibitory effect was alleviated or even converted into an enhancement. For nicks on the TS, regardless of their inhibitory efficiency (high or low), a universal enhancement in Cas12a activation was observed across multiple nicked NTS backgrounds relative to a full NTS (Figure 2b). This effect was most pronounced when the TS was paired with an NTS nicked at internal positions (Figure 2c). This may be attributed to the introduction of nick sites on the NTS, which increases duplex instability, facilitates unwinding, and accelerates R‐loop formation.

Notably, TS nicks with similar inhibitory efficiency exhibited varying levels of Cas12a activation enhancement when paired with a fixed nicked NTS, suggesting that nick sites on both TS and NTS exert synergistic regulation in split activator systems (Figure S3). To more clearly present this synergistic regulation, we divided the split activator into three parts relative to the PAM position for analysis. As illustrated in Figure 2d, proximal nicks in TS exhibited lower Cas12a activation enhancement compared to distal ones. Since the seed sequence in dsDNA activators is crucial for initial recognition and binding of Cas12a, the presence of nick sites in this region significantly controls activation efficiency, relatively weakening the regulatory effect of nicked NTS [21, 22]. In contrast, when the target strand was broken at the distal region, its governance was comparatively weaker in split activators. When nicks were in the distinct region on both the TS and NTS, the enhancing effect of the NTS seemed more pronounced. Long and staggered nick sites on both duplex strands greatly enhance local DNA flexibility, inducing DNA conformational strain to drive Cas12a activation. Therefore, these findings indicate that the combined nicking profile of both strands is not merely additive but exhibits a strong synergy, ultimately dictating the overall efficiency of the Cas12a *trans*‐cleavage process.

**FIGURE 3:**
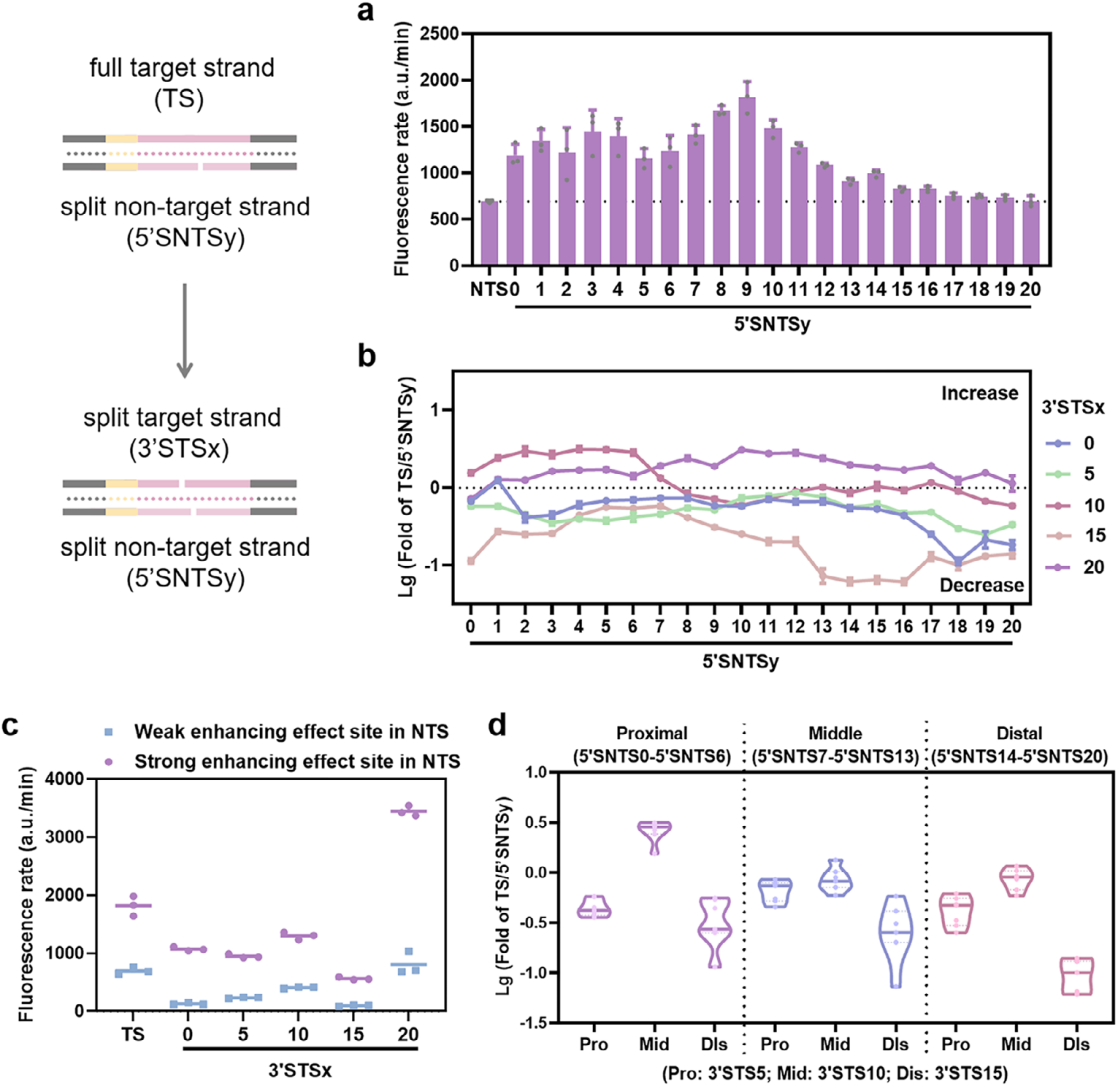
Nicked non‐target strand has its *trans*‐cleavage enhancement effect abolished by broken target strand. (a) Overall enhancement of Cas12a *trans*‐cleavage activity by TS/5’SNTSy. (b) Activity difference of 3’STSx/5’SNTSy relative to TS/5’SNTSy (dashed line = baseline). (c) The effect of NTS nicks on Cas12a activity depends on the specific nick present on the TS. (d) Synergistic control of Cas12a activity by TS and NTS nick positions relative to the PAM. Data represent mean ± SD of three technical replicates; reactions were performed at 37°C for 120 min.

Next, we employed the same methodology to investigate the effect of 5'SNTSy on Cas12a *trans*‐cleavage performance (Figure S4). When paired with an intact TS, nicked NTS generally enhanced Cas12a *trans*‐cleavage activity compared with the full‐length NTS (Figure 3a), consistent with the reduced duplex stability mechanism described above. However, when NTS nicks were combined with nicked TS, the outcomes became position‐dependent (Figure 3b). Most non‐target strands containing nicks showed lower fluorescence rates when paired with split target strands compared to complete target strands, regardless of whether the nicks had strong activation effects or weak activation effects (Figure 3c and Figure S5). Notably, several TS positions with weak inhibitory effects (e.g., 3’STS10 and 3’STS20) still exhibited significant enhancement when paired with nicked NTS. Besides, three parts, derived from split non‐target strands, showed similar enhancing effect paired with TS nicked at different locations (Figure 3d). In particular, the combination of 3′STS11 with 5′SNTS5 yielded up to a 9‐fold enhancement in *trans*‐cleavage activity relative to intact TS/NTS (Figure S2). This strongly suggests that the regulatory function of the NTS itself was modulated by the TS.

Taken together, the activation effect of Cas12a in split activator systems is primarily governed by the TS, while the NTS plays a modulatory role. As the key regulator of Cas12a activation, TS with nick sites may impose constraints on the binding kinetics of the Cas‐crRNA complex, thereby hindering Cas12a activation. Conversely, broken NTS has an indirect effect on Cas12a activation, potentially by reducing the structural stability of the DNA duplex. A systematic investigation into the nicks on the dsDNA activator will enable the identification of strategies to improve Cas12a activation efficiency, facilitating the design of assays with higher sensitivity.

### 2.2 Mechanistic Insights Into Position‐Dependent *Trans*‐Cleavage by Split dsDNA Activators

We further investigated the molecular mechanism for how the nick position in the activator influences its efficiency in activating Cas12a *trans*‐cleavage. As a prerequisite, we examined the integrity of the 3’STSx/5’SNTSy complex, particularly for very short sequences. Melting curve analysis showed that even the complex formed by the shortest single strand exhibited Tm values of 41.5°C, indicating that these complexes remained robustly stable at the reaction temperature (Figure S6a and Figure 6b). PAGE analysis further confirmed that the 3’STSx/5’SNTSy complex could stably exist and thereby activate Cas12a (Figure S6c and Figure 6d).

**FIGURE 4:**
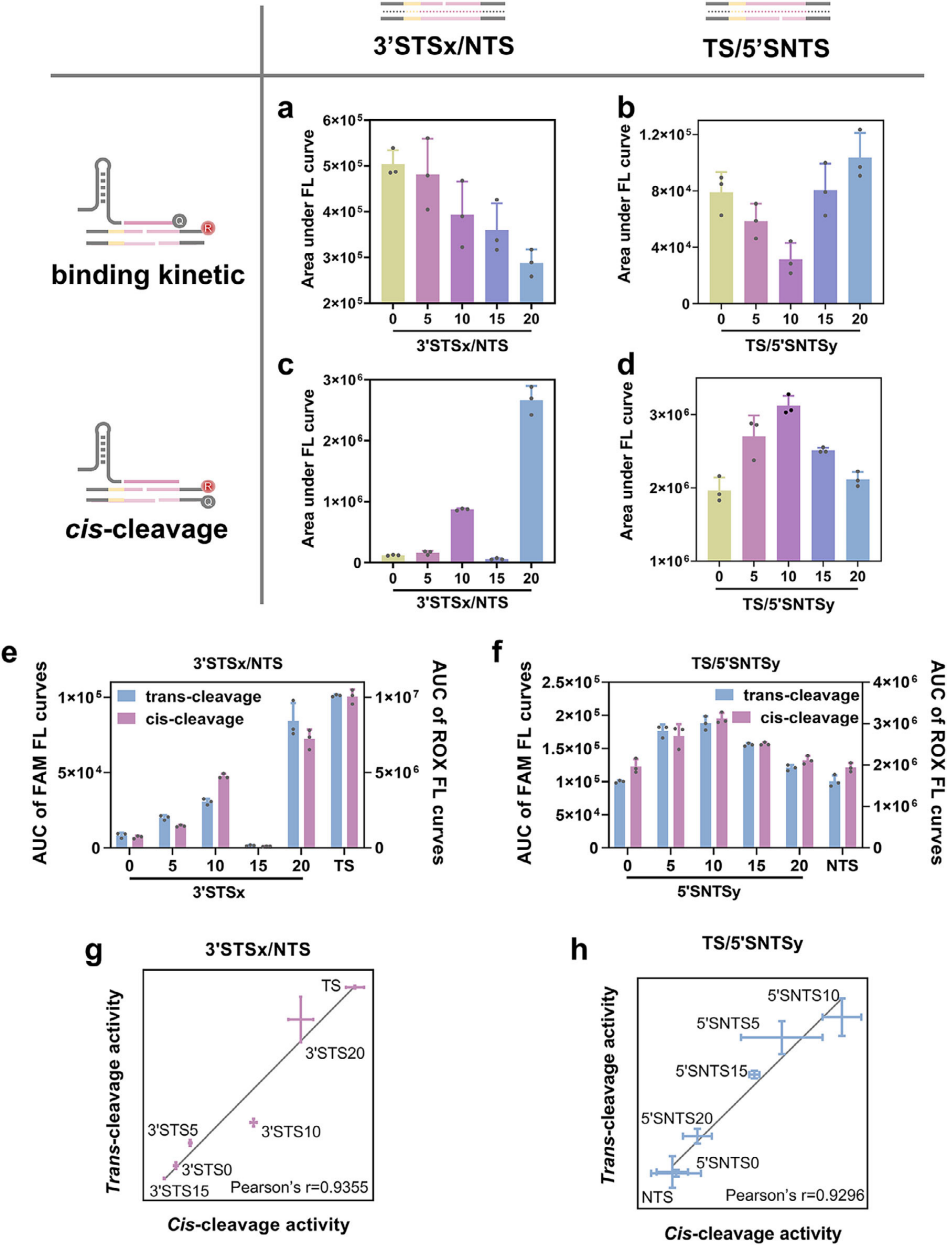
Molecular basis of position‐dependent *trans*‐cleavage activity. (a,b) The analysis of area under the curve for the split activator‐crRNA binding. (c,d) Comparison of *cis*‐cleavage activities based on AUC. (e,f) Area under FL curve of *cis*‐cleavage (ROX) and *trans*‐cleavage (FAM) at (e) 3'STSx/NTS and (f) TS/5'SNTSy. (g,h) Linear correlation between *cis*‐ and *trans*‐cleavage activities of (g) 3'STSx/NTS and (h) TS/5'SNTSy. The data represent mean ± SD of three technical replicates.

Next, we observed the binding between 3’STSx/5’SNTSy and crRNA‐Cas12a ribonucleoprotein (RNP). It is known that Cas12a activation occurs via RNP binding and subsequent *cis*‐cleavage, which exposes the steric site (Figure S7). By modifying the quencher BHQ‐2 at the 3' end of the spacer RNA and the fluorophore ROX at the 5' end of the activator, we established a method for the real‐time monitoring of the first event. During the monitoring of split activator‐RNP binding, the FL curve showed two distinct phases: an initial decrease resulting from binding, followed by a sharp increase caused by *cis*‐cleavage. The presence of nicks on the target strand resulted in a slower decrease in the fluorescence curve compared to the intact one, indicating that nicks indeed impede the binding between the activator and the RNP complex (Figure S8a). The area under FL curve (AUC) serves as a composite measure of the reaction's progression throughout its entire time course. As shown in Figure 4a, the closer the nick position is to the PAM, the higher the observed AUC value, which corresponds to a slower decrease in the fluorescence curve. It seems that nicks proximal to the PAM exert a stronger disruptive effect on the interaction between the activator and the spacer RNA. Conversely, nicks on the non‐target strand exhibited a similar fluorescence trend to the non‐nicked control (Figure S8b). A smaller AUC was observed when the nicks were located near the middle (Figure 4b).

Then, we examined the *cis*‐cleavage activity of Cas12a using a split activator, in which the TS was labeled with ROX and the NTS with BHQ‐2. It was observed that the fluorescence signal underwent a progressive recovery due to the *cis*‐cleavage activity. The intact TS plateaued earlier than its nicked counterpart (Figure S8c), and the overall efficiency of *cis*‐cleavage was dependent on binding, with the binding speed determining its efficiency (Figure 4a,c). An exception was observed at the 3’STS15/NTS, potentially because the nick at this location triggers a conformational change, reducing the affinity or proper orientation of the Cas12a catalytic pocket for the duplex substrate and thus impeding *cis*‐cleavage. Furthermore, a similar trend was observed at the nicked NTS: a lower AUC of binding, indicative of faster binding, correlated with a higher AUC of *cis*‐cleavage, suggesting more efficient cleavage (Figure 4b,d). These results suggest that the activator‐RNP binding event dictates the overall speed of the subsequent reaction cascade, directly controlling the rate at which downstream cleavage events can be initiated.

Previous studies indicated that Cas12a uses a single catalytic site to cleave dsDNA activators via *cis*‐activity prior to non‐specific ssDNA reporters via *trans*‐activity [4, 23]. Efficient cleavage of PAM‐distal dsDNA activators alleviates steric hindrance, facilitating ssDNA reporters to enter the catalytic pocket of Cas12a [24, 25]. We speculated that the speed of *cis*‐cleavage may be the rate‐determining factor for the subsequent *trans*‐cleavage activity. To validate the proposed relationship, we compared the magnitudes of the AUCs resulting from kinetic measurements of *cis*‐ and *trans*‐cleavage. It was shown that coordinated fluorescence changes between *trans*‐ and *cis*‐cleavage occurred, regardless of whether the nicks were in the TS (Figure 4e) or the NTS (Figure 4f). The mechanistic basis for this position‐dependent enhancement became apparent through correlation analysis. This positively coordinated behavior became apparent in linear correlation diagrams (Figure 4g,h). Combinations of TS and NTS nicks likewise exhibited similar trends of fluorescence change and a positive correlation between *trans*‐ and *cis*‐cleavage activities (Figure S9). Collectively, these findings suggest that the location of nicks impacts the accessibility of the Cas12a catalytic pocket by causing allosteric changes in the duplex. This altered accessibility is reflected in the rate of *cis*‐cleavage, thereby indirectly influencing the *trans*‐cleavage reaction. Acting as a kinetic bottleneck, the *cis*‐cleavage step restricts the overall output of the *trans*‐cleavage reaction.

### 2.3 Universal Strategy for Sensitivity Detecting microRNA Using Split dsDNA Activator

**FIGURE 5:**
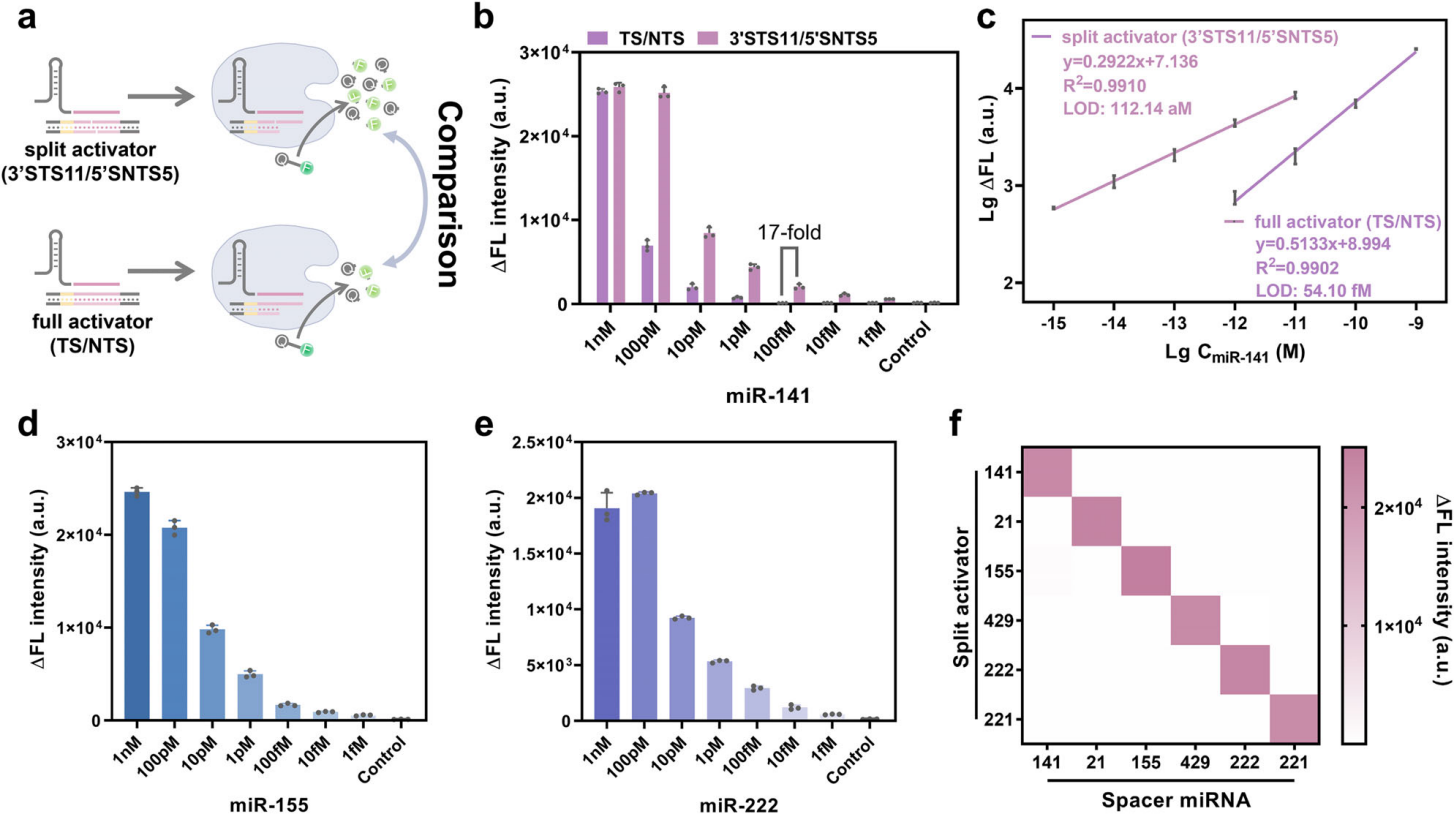
Highly Sensitive miRNA Detection Using Split Activator. (a) Comparison between 3’STS11/5’SNTS5 and TS/NTS on Cas12a‐mediated miRNA detection. (b) Fluorescence enhancement of split and full dsDNA activators as a function of miR‐141 concentrations. (c) Linear relationship between log (signals) and log(miR‐141 concentration) using 3’STS11/5’SNTS5 or TS/NTS. (d,e) Fluorescence increase of 3’STS11/5’SNTS5 in response to different concentrations of d miR‐155 and e miR‐222. (f) Heatmap compares the fluorescence increase of *trans*‐cleavage activity for different miRNAs tested with different split activators. ΔFL intensity = FL_120 min_—FL_0 min_. Reactions were incubated for 120 min at 37°C. The data represent mean ± SD of three technical replicates.

Having systematically mapped the position‐activity landscape and elucidated the mechanistic basis, we next exploited these design principles to engineer split activators for practical biosensing applications. We prioritized RNA detection as our initial target, given the critical role of RNA biomarkers in disease diagnosis and the technical challenges associated with their sensitive detection [26, 27]. Using miR‐141 as a representative biomarker, we compared the detection sensitivity of the split dsDNA activator and the full dsDNA activator guided by target miRNA in the Cas12a system (Figure 5a). Real‐time fluorescence signals of Cas12a‐mediated miR‐141 detection activated by split and full dsDNA activators were monitored at various concentrations ranging from 10 fM to 1 nM (Figure S10). As shown in Figure 5b, comparable activation performance was observed between the two activators at high miR‐141 concentrations, whereas significant signal divergence emerged at low target concentrations. In the Cas12a system, the employment of split dsDNA activators achieved an ultrasensitive miR‐141 detection (limit of detection LOD: 112 aM), with a 480‐fold higher sensitivity than conventional full dsDNA activators (Figure 5c).

Building on the flexibility of spacer RNA replacement in a split crRNA system, we next assessed the generalizability of this strategy. Two additional cancer miRNA biomarkers (miR‐155 and miR‐222) yielded analogous detection sensitivity to miR‐141, highlighting the ability of split dsDNA activators to universally improve sensitivity (Figure 5d,e and Figure S11). Moreover, we evaluated the selectivity of split dsDNA activators across multiple miRNAs. Our results showed that strong fluorescence signals occurred exclusively when split dsDNA activator paired with its perfectly matched miRNA target, indicating the high detection specificity of Cas12a activated by split dsDNA activators (Figure 5f and Figure S12). All these data demonstrated the superior miRNA sensing performance of Cas12a activated by split dsDNA activators.

### 2.4 A Novel Split Activator‐based CRISPR Platform for High‐Performance miRNA Imaging in Cells

**FIGURE 6:**
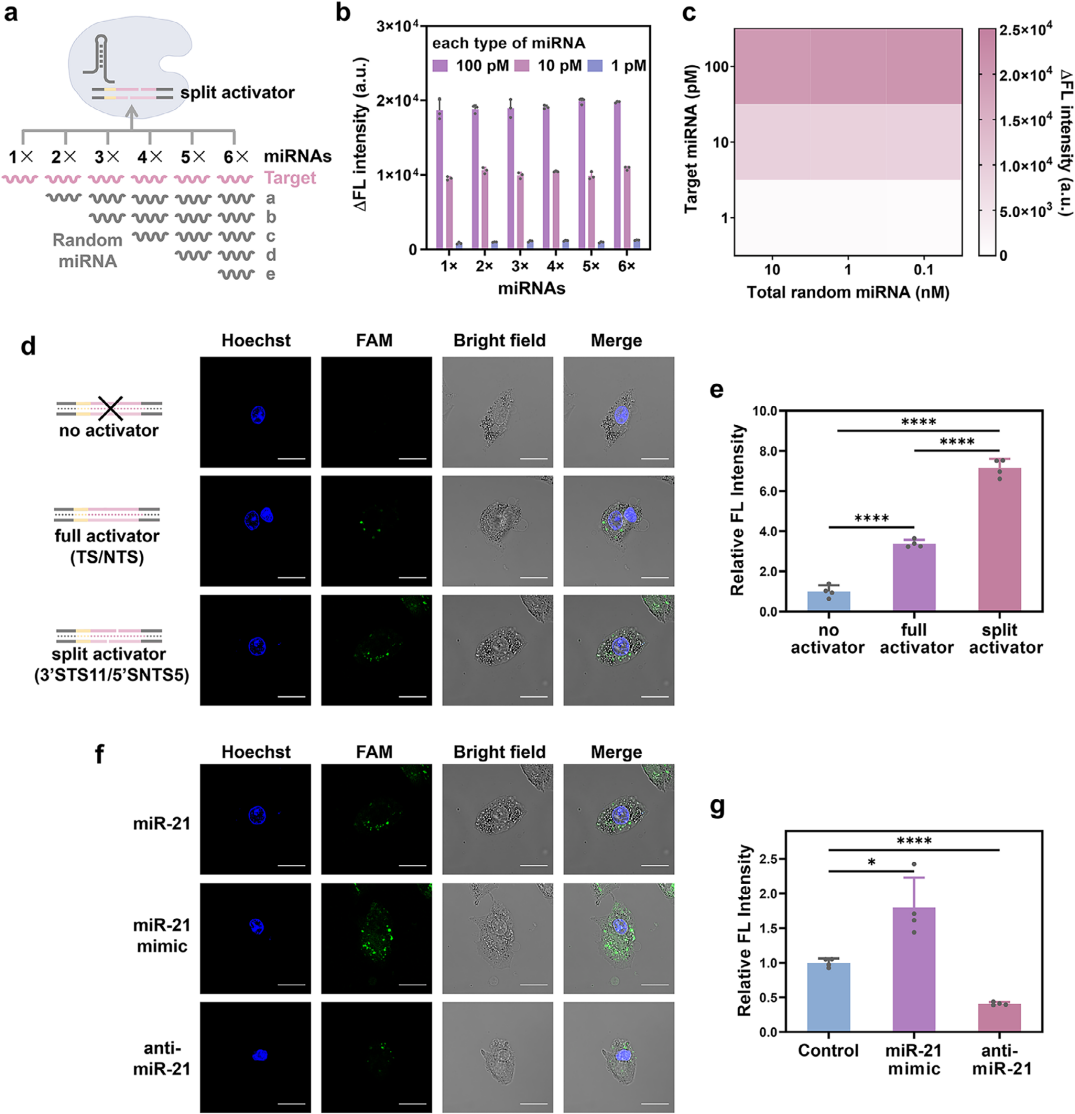
Evaluation of split activator platform in intracellular miRNA detection. (a) Schematic of doping target miRNA with random miRNAs to simulate a complex biological environment. (b) Fluorescent response of target miRNA mixed with different types of random miRNA under different concentration of miRNAs. (c) Assessment of interference on signal output from different concentrations of random miRNA. ΔFL intensity = FL_120 min_ – FL_0 min_. Reactions were incubated for 120 min at 37°C. The data represents mean ± SD of three technical replicates. (d) Confocal fluorescence images and (e) relative fluorescence intensity of miR‐21 detection in HepG2 cells incubated with split crRNA‐guided Cas12a system. (f) Confocal fluorescence images and (g) relative fluorescence intensity of HepG2 cells pretreated with miR‐21 mimics or anti‐miR‐21, using the Cas12a system activated by split activator. Scale bars = 20 µm. The data represent mean ± SD of four technical replicates. Statistical significance was determined by *t*‐test (*p < 0.05; **p < 0.01; ***p < 0.001; ****p < 0.0001.).

Endogenous RNAs pose a major challenge to Cas12a‐based detection, as they competitively bind to the crRNA scaffold. Employing the CRISPR system in excess guarantees that target detection remains unaffected by other RNAs. Using miRNA detection as a model, we performed interference resistance validation through mixing target miRNA with random miRNAs to evaluate the detection capability of an excess amount of the Cas12a system under competitive conditions (Figure 6a). Consistent with prior studies [28], as shown in Figure S13, the rate of fluorescence generation decreased with the increasing amount of random miRNA added. Significant competitive inhibition was observed when the total miRNA concentration exceeded that of the Cas12a system. While the Cas12a system was in excess relative to the total miRNA, the increase in random miRNA had a negligible impact on target miRNA detection (Figure 6b and Figure S14). The fluorescence kinetic curves were similar despite the addition of random miRNA. The detection of the target miRNA is impervious to even an overwhelming excess of random miRNAs (up to 100–10000 fold), provided that the Cas12a system is in excess (Figure 6c). The mechanism underpinning this robustness is the provision of a surplus of spacer binding sites by excess Cas12a, which effectively eliminates competitive inhibition and safeguards the efficiency of target detection.

Encouraged by the outstanding sensing capability of split activator and the elimination of competitive inhibition by the excess Cas12a system in vitro, we investigated the performance of miRNA detection in cells using the split crRNA‐guided Cas12a system. We chose HepG2 cells, with a high expression of miR‐21 [29], as a model to explore the intracellular sensing capacity. As shown in Figure 6d, confocal laser scanning microscope (CLSM) imaging revealed that strong intracellular fluorescence was observed in cells treated with split crRNA‐guided Cas12a system activated by full activator and split activator. By contrast, cells lacking the activator showed no obvious intracellular fluorescence, indicating that the Cas12a system was not activated. Split activator‐treated cells yielded approximately 2.2‐fold enhancement of fluorescence intensity relative to full activator, further confirming the superior detection efficiency of split activator (Figure 6e). Meanwhile, an increased fluorescence signal was observed in live HepG2 cells upon addition of miR‐21 mimics, while a feeble fluorescence signal was detected in cells pretreated with anti‐miR‐21 (Figure 6f,g). These results demonstrated that the fluorescence originated specifically from target miR‐21, confirming the high specificity of the split crRNA‐guided Cas12a system. Furthermore, a similar imaging effect was observed for the detection of miR‐141 in HepG2 cells, demonstrating its capability for practical application in imaging multiple intracellular miRNAs (Figure S15). To determine whether the observed fluorescence reflects endogenous miRNA abundance, we compared Cas12a‐based detection with the gold standard qRT‐PCR by miRNA mimic titration (Figure S16a,b). A strong linear correlation (R^2^ = 0.9697) was observed between the two methods (Figure S16c), confirming that the Cas12a signal is indeed correlated with miRNA abundance. Additionally, the CCK‐8 assay showed that no significant reduction in viability was observed under our experimental conditions (Figure S17).

### 2.5 Multiplex Profiling of Intracellular miRNAs With Split Activator‐Based Cas12a System for Cancer Cell Identification

**FIGURE 7:**
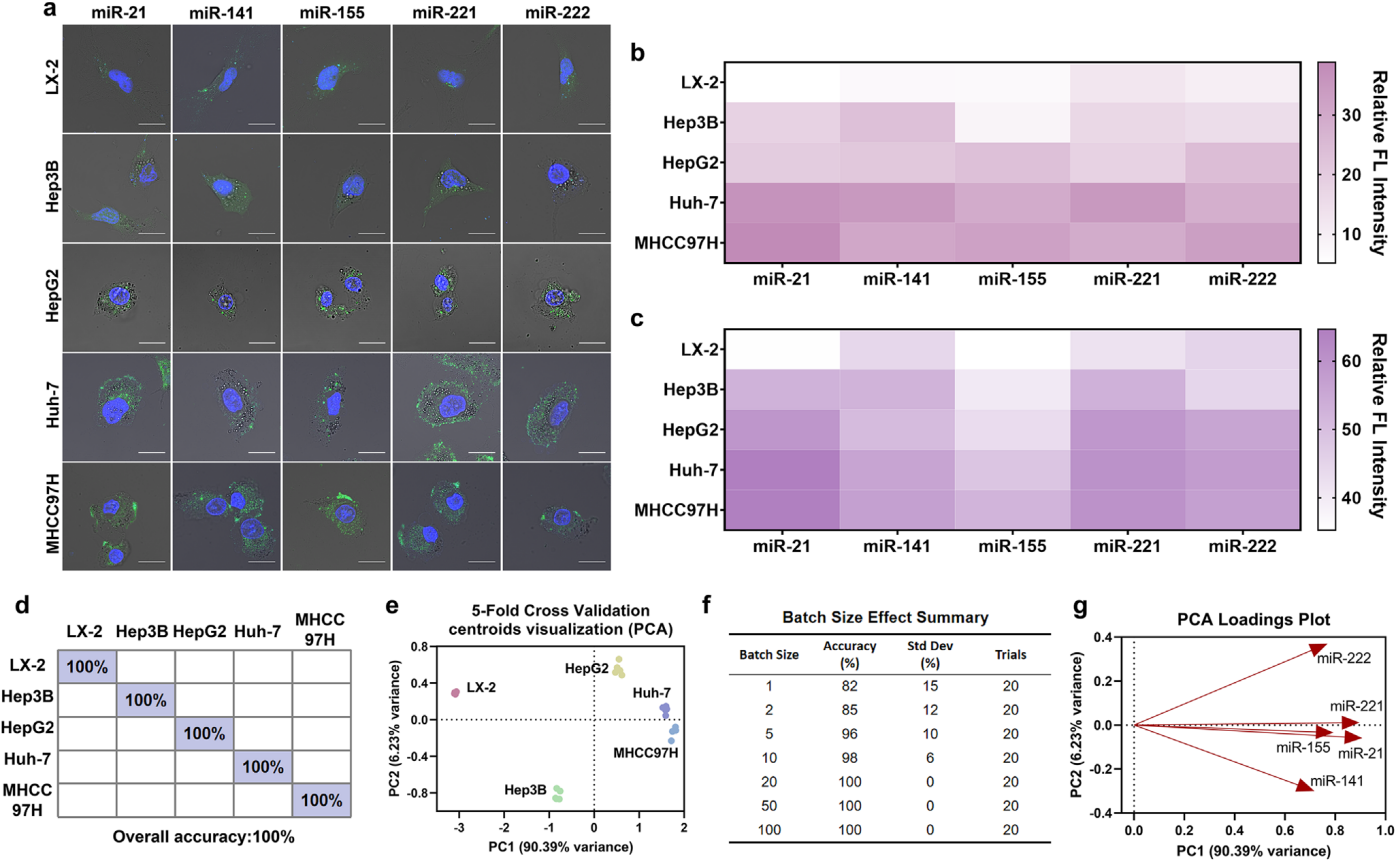
MiRNA signature analysis in a panel of cell lines, enabling precise classification via the k‐NN algorithm. (a) Confocal microscopic image of various miRNA in liver cell lines of varying aggressiveness. These liver cell lines included one normal hepatic cell line (LX‐2) and four liver cancer cell lines with distinct malignant potential: highly malignant (MHCC97H [38, 39]), moderately malignant (Huh‐7 [40, 41]), and low malignant (HepG2 and Hep3B [40, 41]). (b,c) Relative fluorescence intensity quantified from (b) confocal images and (c) flow‐cytometry data (normalized to untreated control). (d) Performance of classifying liver cancer cell lines based on the k‐NN algorithm. (e) Classification performance visualized in 2D PCA space. Each point represents the centroid of a specific cell type from one fold in a five‐fold cross‐validation. (f) Classification accuracy across different batch sizes (*n* = 20 trials). (g) PCA loading plot of five miRNAs along PC1 and PC2.

The utility and versatility of the split crRNA‐guided Cas12a system were further assessed by applying it to the detection of multiple miRNAs across a range of liver cell types with varying differentiation statuses. Previous studies have shown that some specific miRNAs, including miR‐21, miR‐155, miR‐221, miR‐222 or miR‐141, are differentially expressed in hepatocellular carcinoma (HCC) when compared to normal counterparts. Moreover, their expression levels are also associated with the initiation and progression of HCC [30, 31, 32, 33, 34]. As shown in Figure 7a and Figure S18, the split crRNA‐guided Cas12a system successfully imaged multiple specific miRNAs in various cell lines. Flow cytometry data revealed a gradual increase in FAM intensity consistent with elevated malignancy (Figure S19). These results align with previous findings and further support the progressive association between miRNA dysregulation and the degree of cellular malignancy. The data presentation in heatmaps, based on intracellular fluorescence quantification and flow cytometry, showed that certain miRNAs enabled effective discrimination between cancerous and normal cells. However, achieving an absolute distinction between these five liver cell lines of differing aggressiveness was not possible with any one miRNA tested above (Figure 7b,c).

To overcome the limitation of single‐biomarker analysis, we leveraged multivariate profiling of the five miRNAs to enable precise classification of liver cell lines with varying malignant potential [35, 36, 37]. The K‐nearest neighbors (k‐NN) algorithm was employed to parse flow cytometry datasets, integrating the five miRNA features through 5‐fold cross‐validation combined with a batch averaging strategy. This machine learning‐driven approach achieved 100% classification accuracy on the test set (Figure 7d), with stable, well‐separated clustering of cross‐validation centroids in PCA space confirming robust discrimination among cell lines of different aggressiveness (Figure 7e). Classification performance exhibited strong batch‐size dependence: prediction accuracy improved progressively from 82.0% ± 15.4% for individual samples to a stable 100% with batch sizes ≥20 (Figure 7f), demonstrating the noise‐suppression effect of ensemble averaging. PCA loading analysis identified miR‐21, miR‐221, and miR‐155 as the primary discriminative features, with their dominant contributions consistent with established oncogenic roles [30, 31, 32] (Figure 7g). These results demonstrate that multiplexed miRNA profiling combined with machine learning enables accurate cell malignancy stratification—a capability unattainable through single‐biomarker approaches.

### 2.6 Amplification‐Free Long RNA Detection Enabled by Split Activators

Long RNA targets present unique challenges in CRISPR‐based detection. While short RNAs such as microRNAs (∼22 nt) are readily detected, longer RNA sequences often require pre‐amplification steps such as reverse transcription PCR (RT‐PCR) or recombinase polymerase amplification (RPA). These additional procedures increase assay complexity, cost, and turnaround time, creating barriers for point‐of‐care applications. Having demonstrated that split activators achieve superior performance in short RNA detection, we next investigated whether this enhancement extends to longer RNA targets. We employed a 100‐nucleotide fragment of bacteriophage MS2 RNA as a model target. MS2 serves as a widely used surrogate for hazardous human viruses in evaluating water treatment and air purification efficacy, making its sensitive detection of significant practical importance.

**FIGURE 8:**
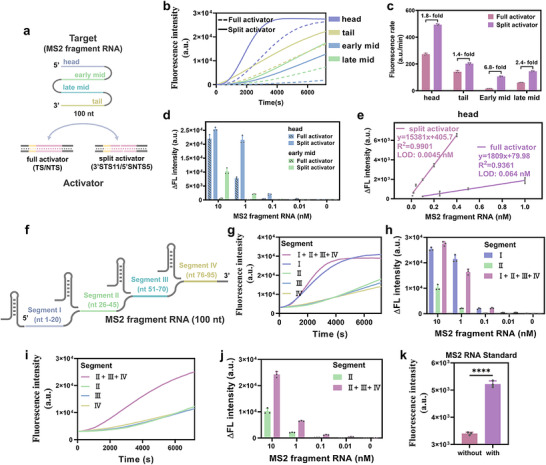
Amplified detection of long‐sized RNA via a split activator mechanism. (a) Illustration of MS2 RNA target bound by activators at various positions. (b,c) Comparison of TS/NTS and 3’STS11/5’SNTS5 efficacy on the MS2 RNA end region. (d) Sensitivity analysis for MS2 RNA detection using TS/NTS and 3’STS11/5’SNTS5 across the head and early mid regions. (e) Linear response of MS2 RNA detection using TS/NTS and 3’STS11/5’SNTS5‐based system. (f) Illustration of MS2 fragment RNA target bound by activators at four positions. (g,h) Comparing (g) fluorescence curves and (h) dose‐response signals of single and four combinatorial Activators. (i,j) Illustration of three MS2‐activator complexes, with detection performance comparison of single versus three multiplexed activators. (k) Detection assay for authentic MS2 RNA standards. The data represent mean ± SD of three technical replicates. Statistical significance was determined by t‐test (*p < 0.05; **p < 0.01; ***p < 0.001; ****p < 0.0001.).

We systematically evaluated the performance of split activators (3'STS11/5'SNTS5) versus full‐length activators (TS/NTS) across four distinct positions spanning the MS2 RNA fragment: two terminal regions (5' and 3' ends) and two internal regions (early and late mid segments) (Figure 8a). When targeting terminal regions, split activators yielded 1.4‐ to 1.8‐fold enhancement in *trans*‐cleavage activity compared to full‐length counterparts. Remarkably, internal regions exhibited dramatically stronger enhancement, with split activators achieving 2.4‐ to 6.8‐fold increases in fluorescence rates (Figure 8b,c). This pronounced position‐dependent effect suggests that accessibility constraints at internal positions—potentially arising from steric hindrance or local sequence context (Figure S20)—are more effectively overcome by split activators. The enhanced activity translated directly into improved detection sensitivity. Dose‐response analysis revealed that split activators enabled detection of MS2 RNA at concentrations as low as 4.5 pM for optimal internal regions and 20–40 pM for terminal regions (Figure 8d,e). In contrast, full‐length activators targeting the same region achieved a detection limit of 64 pM, which represents a 14‐fold improvement with split activators and eliminating the need for pre‐amplification (Figure 8e). Excellent linear responses were obtained across the tested concentration range (Figure S21), demonstrating robust quantitative performance. This position‐dependent sensitivity enhancement underscores the importance of rational target site selection for maximizing detection performance.

We next investigated whether this amplified benefit was translatable to the context of longer RNA detection. It is known that long RNA molecules can hinder detection by forming complex secondary structures that create steric hindrance, thereby impeding the access of Cas proteins. We sought to boost the sensitivity of our assay through the concurrent detection of multiple regions within the long RNA. As illustrated in Figure 8f, four segments within a synthetic fragment of the MS2 RNA were designed for simultaneous detection. The results in Figure 8g,h showed that the simultaneous detection of all four fragments displayed similar fluorescence kinetics to the Segment I assay, with both producing a detectable signal at the lowest tested concentration of 0.01 nM. The apparent masking effect may stem from the 5' end location of segment I facilitating Cas protein access, which causes its strong signal to overshadow those of other segments. While terminal regions might be easier to access, detecting internal sequences is more meaningful for viral RNA genomes, as their protein‐coding regions are predominantly harbored within these areas. For the internal regions of the MS2 fragment RNA, simultaneous detection of three regions enabled faster fluorescence growth and a lower detectable concentration compared to the detection of a single region (Figure 8i,j). Furthermore, this multi‐region simultaneous strategy proved capable of reliably detecting practical MS2 RNA standards in the presence of four different types of split activators (Figure 8k). This success validates the efficacy of our multi‐region targeting method and highlights its general utility in overcoming structural barriers for nucleic acid sensing, establishing a key step toward more reliable long RNA diagnostics.

### 2.7 Programmable Activation of Universal Cas12a via a Split Designer Activator for Multiplex Molecule Detection

**FIGURE 9:**
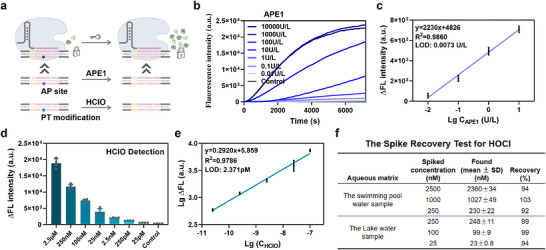
Multiplexed biosensing via programmable split activator system. (a) Schematic of split designer activator for multiplex molecule detection. Small molecule HClO or APE1 enzyme cleaves at a specific modified site on the activator to activate Cas12a. (b) Fluorescence curves of APE1 detection at different concentrations. (c) The linear relationship of APE1 detection method. (d) FL increment for detecting HClO at different concentrations. (e) Linearity of HClO detection over a concentration range of 100 nM–25 pM. (f) The recovery rate of the HOCl detection. The data represent mean ± SD of four technical replicates.

The programmable nature of split activator positions, which can either suppress or enhance Cas12a activity, provides a versatile framework for detecting diverse molecular targets beyond nucleic acids. By incorporating target‐responsive cleavable sites into the activator backbone, we demonstrate the platform's extensibility to enzyme and small molecule detection. The initial break of dsDNA activator at 3'STS15 keeps the Cas12a system off. A subsequent break at 5'SNTS5 on the NTS strand, triggered by a target, turns the system on by reactivating Cas12a and restoring fluorescence (Figure 9a).

For APE1 enzyme detection, we designed the AP site into the NTS strand. Human apurinic/apyrimidinic endonuclease 1 (APE1) is a key enzyme in the base excision repair pathway, cleaving AP sites in DNA. Its overexpression in cancers, which promotes tumor development, makes it a valuable diagnostic target [42, 43]. As shown in Figure 9b, the addition of APE1 cleaves the AP site, which in turn activates Cas12a's *trans*‐cleavage, leading to the restoration of fluorescence. The expected cleavage fragments were clearly resolved by urea PAGE following APE1 treatment (Figure S22). This method sensitively detects APE1 with excellent linearity (LOD = 0.0073 U/L, Figure 9c). These results illustrate the flexibility of this strategy for detecting APE1 enzyme.

To detect small molecule HClO, we incorporated a phosphorothioate (PT) modification into the activator. The overuse of hypochlorous acid (HClO), a common disinfectant, leads to aquatic contamination, where it forms toxic byproducts that endanger ecosystems and public health. Therefore, monitoring is essential. Notably, HClO cleaves DNA specifically at PT sites [7]. Upon its cleavage by HClO, the Cas12a system is activated and generates a measurable fluorescent response. Urea PAGE validated the expected HClO cleavage products (Figure S22). Figure 9d showed that this method can detect HClO at concentrations as low as 25 pM. The log‐log plot of fluorescence increment as a function of HClO concentration reveals a linear relationship across the range of 100 nM to 25 pM. The limit of detection was determined to be 2.37 pM based on the 3σ/k criterion (Figure 9e). To evaluate practical application, we tested our system on swimming pool and lake water samples. The HClO concentration reached 19.5 µM in swimming pool water, while it was 0.31 µM in lake water. Spike‐recovery tests across three concentrations yielded excellent recovery rates of 92–103% (pool) and 94–99% (lake), demonstrating high accuracy and robustness in complex matrices (Figure 9f).

In summary, the split activators effectively enhance the sensitivity of the Cas12a system for detecting nucleic acid targets, subsequently extending its capability through functional modifications to rapidly and sensitively detect non‐nucleic acid targets. From a practical application perspective, this conformational engineering of activators paves the way for applying CRISPR technology across multiple fields, promising an expanded recognition of the CRISPR technology's application potential.

## 3 Discussion

To systematically decode the position‐activity relationship of split Cas12a activators, we constructed a comprehensive regulatory atlas by systematically mapping over 200 split configurations. While previous studies relying on sparse sampling reported inconsistent effects, our combinatorial screening revealed a clear position‐dependent landscape: target strand (TS) nicks generally suppress activity, whereas internal non‐target strand (NTS) nicks significantly enhance it. Crucially, we identified non‐additive synergistic pairings, with the 3'STS11/5'SNTS5 combination achieving exceptional activation enhancement. These findings provide the “programmable control” over *trans*‐cleavage efficiency sought in the introduction, demonstrating that optimal performance requires exploring the full design space rather than relying on isolated modifications.

Translating these mechanistic insights into diagnostic performance, the optimized split activators overcame the sensitivity limitations of conventional amplification‐free methods. We achieved attomolar detection of microRNAs (LOD: 112 aM) directly, representing a ∼480‐fold improvement over intact activators and eliminating the need for complex pre‐amplification steps. This enhanced sensitivity, coupled with an excess Cas12a strategy to mitigate cellular RNA interference, enabled the precise profiling of five cancer‐associated miRNAs in live cells. The successful integration of this data with machine learning for highly accurate cell malignancy stratification highlights the platform's potential for high‐precision clinical diagnostics.

Beyond nucleic acids, the versatility of this position‐activity framework was validated through the detection of non‐nucleic acid targets. By engineering target‐responsive cleavable sites at functionally critical positions, we extended the system to detect APE1 enzyme and small molecule HClO with high sensitivity. Collectively, this work transforms the split activator from an empirical observation into a rationally engineerable component. By establishing generalizable design rules, we provide a robust foundation for developing versatile and high‐performance CRISPR‐Cas12a biosensors capable of diverse analytical applications, from molecular diagnostics to environmental monitoring.

## 4 Methods

### 4.1 Chemicals and Materials

All oligonucleotides used in this study (sequences in Supplementary Table S1 and S2) were synthesized and purified by Sangon Biotech Co., Ltd (Shanghai, China) and Generay Biotechnology Co., Ltd. (China). The LbaCas12a (Cat No. CAS‐12B‐100) protein was expressed and purified by EZassay Biotech (Shenzhen, China). 10× buffer r2.1 (Cat No. B6002V), 10× buffer r1.1 (Cat No. B6001V), and APE1 enzyme (Cat No. M0282S) were purchased from New England Biolabs Inc. (Beijing, China). LipofectamineTM 3000 (lipo, Cat No. L3000015), serum reducing medium (Opti‐MEM, Cat No. 31985062) were purchased from Thermo Fisher Scientific Inc. (USA). Dulbecco's Modified Eagle's Medium (DMEM, Cat No. MA0212), fetal bovine serum (Cat No. PWL217), 0.25% trypsin (Cat No. MA0233), Phosphate‐buffered saline (PBS, Cat No. MA0015), and penicillin‐streptomycin (P/S, Cat No. MA0110) were purchased from MeilunBio Reagent Co., Ltd (Dalian, China). Hoechst 33342 (Cat No. BL1145B) were purchased from Biosharp Biotechnology Co., Ltd. (China). Sodium hypochlorite aqueous solution (Cat No. 767472) were purchased from Macklin Chemical Reagent Co., Ltd (Shanghai, China).

### 4.2 Preparation of Split dsDNA Activator

To obtain the split dsDNA activator, four single‐stranded, corresponding to the PAM‐proximal and PAM‐distal regions of both the target and non‐target strands, were mixed in a 1:1:1:1 molar ratio and annealed in an annealing buffer (10 mM Tris‐HCl, 90 mM NaCl, pH 7.5). Following denaturation at 95°C for 5 min, the mixture was slowly cooled to 25°C. And the prepared activators were stored at 4°C prior to use.

### 4.3 Evaluate Split Activator Impact on Cas12a *trans*‐Cleavage

A 20 µL reaction mixture containing 1× Buffer r2.1, 25 nM scaffold RNA, 0.5 nM spacer RNA, 250 nM ssDNA reporter (F‐Q), and 25 nM Cas12a was prepared. The reaction was then initiated by adding 25 nM split dsDNA activator. The fluorescence intensity of the reaction solution was monitored in real‐time on Archimed X4 qPCR (RocGene, Beijing) at 37°C, with the FAM channel. All experiments were performed with three independent replicates.

### 4.4 Explore the Binding Effect Between Split Activator and RNP Complex

A 20 µL reaction mixture containing 1× Buffer r2.1, 50 nM scaffold RNA, 50 nM spacer RNA labeled with BHQ2, and 50 nM Cas12a was prepared. The reaction was then initiated by adding 50 nM split dsDNA activator labeled with ROX. The fluorescence intensity of the reaction solution was monitored in real‐time on Archimed X4 qPCR (RocGene, Beijing) at 37°C, with the ROX channel. All experiments were performed with three independent replicates.

### 4.5 Assess the Functional Impact of *cis*‐Cleavage on *trans*‐Cleavage

The split dsDNA activator was labeled with ROX and BHQ2 at the PAM‐distal ends of the target and non‐target strands, respectively. A 20 µL reaction mixture containing 1× Buffer r2.1, 50 nM scaffold RNA, 50 nM spacer RNA, 250 nM ssDNA reporter (F‐Q), and 50 nM Cas12a was prepared. The reaction was triggered by introducing 50 nM of the modified split dsDNA activator. The fluorescence intensity of the reaction solution was monitored in real‐time on Archimed X4 qPCR (RocGene, Beijing) at 37°C, with the ROX and FAM channels. All experiments were performed with three independent replicates.

### 4.6 Amplification‐Free RNA Detection Enabled by A Split Activator Strategy for Cas12a

For miRNA detection, a 20 µL reaction mixture containing 1× Buffer r2.1, 25 nM scaffold RNA, 25 nM dsDNA activator, 250 nM ssDNA reporter (F‐Q), and 25 nM Cas12a was prepared. The reaction was then initiated by adding miRNA with various concentrations. The fluorescence intensity of the reaction solution was monitored in real‐time on Archimed X4 qPCR (RocGene, Beijing) at 37°C, with the FAM channel.

For long‐sized RNA detection, a 20 µL reaction mixture containing 1× Buffer r2.1, 50 nM scaffold RNA, 50 nM dsDNA activator, 250 nM ssDNA reporter (F‐Q), and 50 nM Cas12a was prepared. The reaction was then initiated by adding long‐sized RNA at various concentrations. The fluorescence intensity of the reaction solution was monitored in real‐time on Archimed X4 qPCR (RocGene, Beijing) at 37°C, with the FAM channel. All experiments were performed with three independent replicates.

### 4.7 Assessing Detection Performance in a Complex miRNA Environment

A 20 µL reaction mixture containing 1× Buffer r2.1, 25 nM scaffold RNA, 25 nM dsDNA activator, 250 nM ssDNA reporter (F‐Q), 25 nM Cas12a, and a mixture of non‐target miRNAs (types: miR‐222, miR‐21, miR‐221, miR‐155, miR‐429) was prepared. The reaction was then initiated by adding miRNA‐141. The fluorescence intensity of the reaction solution was monitored in real‐time on Archimed X4 qPCR (RocGene, Beijing) at 37°C, with the FAM channel. All experiments were performed with three independent replicates.

### 4.8 Monitoring miRNA via a Split Activator‐Based Sensor in a Complex Cellular Background

The method for cellular delivery of the Cas12a detection system was established according to the previous report with minor modifications [44]. All cells were seeded in a 96‐well plate containing 100 µL culture medium. A 10 µL mixture containing 50 nM scaffold RNA, 50 nM dsDNA activator, 250 nM ssDNA reporter (F‐Q), and 50 nM Cas12a was prepared. Then, the mixture was pre‐incubated with 0.6 µL of lipo3000 dispersed in 100 µL Opti‐MEM at room temperature for 20 min, followed by adding into the 96‐well plate for 2 h. After incubation, all cells were washed with PBS, and 50 µL Hoechst 33342 was added. FV3000 confocal laser scanning microscopy (Olympus, Japan) was employed to image the cells, and the data analysis software (FV31S‐SW) was used to analyze the fluorescence data extracted from the images. For the modulation assays, anti‐miRNA and miRNA mimics were treated with cells for 2 h before transfection, respectively.

To perform a flow cytometry experiment, all cells were seeded in a 6‐well culture plate with 2 mL of culture medium per well. Subsequently, the cells were transfected with the split activator system and incubated for 2 h. After incubation, the cells were rinsed twice with PBS, detached from the culture plate using trypsin, and centrifuged at 1000 rpm for 10 min before being suspended in PBS. Flow cytometric analysis was performed using a BD Canto to measure the fluorescence intensity emanating.

To perform the qRT‐PCR experiment, HepG2 cells were seeded in a 96‐well culture plate. The cells were transfected with varying concentrations of miRNA‐141 mimics using lipo3000, followed by incubation for 4 h. After transfection, the cells were subjected to three freeze‐thaw cycles to obtain cell lysates. Before qRT‐PCR analysis of miRNA‐141, the cell lysates (2 µL) was subjected to reverse transcription using the miRNA first Strand cDNA Synthesis Kit (Yeasen, China) to obtain cDNA. PCR reactions were performed using miRNA Universal qPCR SYBR Master Mix (Yeasen, China). The reactions were carried out in a quantitative PCR instrument with the following protocol: an initial denaturation at 95°C for 10 min, followed by 40 cycles of denaturation at 95°C for 15 s, annealing at 60°C for 20 s.

### 4.9 Machine Learning‐Based Cell Classification

Flow cytometry data from five miRNA targets (miR‐21, miR‐141, miR‐155, miR‐221, miR‐222) across five liver cell lines (LX‐2, HepG2, Hep3B, Huh‐7, MHCC97H) were used for machine learning analysis. The K‐nearest neighbors (k‐NN) algorithm was implemented in Python using the scikit‐learn library. For each cell line, fluorescence intensity values from the five miRNAs were normalized and used as feature vectors. where 80% of the samples were used for training and 20% for testing in each fold. Principal component analysis (PCA) was performed using scikit‐learn to visualize clustering patterns and identify key discriminative features. Classification accuracy was calculated as the percentage of correctly classified samples. PCA loading values were analyzed to determine the contribution of individual miRNAs to cell line discrimination. The source code for this project has been made publicly available on GitHub (https://github.com/moontang840/cellclassifier.git).

### 4.10 Simultaneous RNA Sensing With an Engineered Cas12a Split Activator Platform

A 20 µL reaction mixture containing 1× Buffer r2.1, 25 nM scaffold RNA, 250 nM ssDNA reporter (F‐Q), 25 nM Cas12a, and a mixture of split activators targeting distinct miRNAs was prepared. The reaction was triggered with a pool of miRNAs (types: miR‐222, miR‐21, miR‐221, miR‐155, miR‐141). The fluorescence intensity of the reaction solution was monitored in real‐time on Archimed X4 qPCR (RocGene, Beijing) at 37°C, with the FAM channel. To perform the cellular content assay, cell lysates were obtained through freeze‐thaw cycles, and the soluble fraction was collected for analysis [29]. The cell extracts were added to pre‐prepared reaction mixture in a 200 µL test tube. The fluorescence intensity of the reaction solution was monitored in real‐time on Archimed X4 qPCR (RocGene, Beijing) at 37°C, with the FAM channel.

For long‐sized RNA detection, a 20 µL reaction mixture containing 1× Buffer r2.1, 50 nM scaffold RNA, 250 nM ssDNA reporter (F‐Q), 50 nM Cas12a, and a mixture of 50 nM split activators targeting different regions of longer RNA was prepared. The reaction was then initiated by adding long‐sized RNA. The fluorescence intensity of the reaction solution was monitored in real‐time on Archimed X4 qPCR (RocGene, Beijing) at 37°C, with the FAM channel. All experiments were performed with three independent replicates. As to MS2 RNA standards (8.4×10^6^ copies/mL), we first induced self‐cleavage by treatment with high concentrations of magnesium ions (50 mM Tris‐HCl, 30 mM MgCl_2_, pH 7.8) at 95°C for 20 min. Subsequently, this mixture was added to the Cas12 reaction solution. (1× Buffer r2.1, 50 nM scaffold RNA, 250 nM ssDNA reporter (F‐Q), 50 nM Cas12a, and a mixture of 50 nM split activators). The fluorescence intensity of the reaction solution was monitored in real‐time on Archimed X4 qPCR (RocGene, Beijing) after 24 h, with the FAM channel.

### 4.11 APE1 Detection

In a 5 µL reaction mixture, APE1 enzyme at various concentration were mixed with 100 nM AP‐modified dsDNA activator, and then incubated in 1× Buffer r1.1 at 37°C for 2 h. A 15 µL reaction mixture (1× Buffer r2.1, 25 nM scaffold RNA, 0.5 nM spacer RNA, 250 nM ssDNA reporter, 25 nM Cas12a) was added to the mixture. The fluorescence intensity of the reaction solution was monitored in real‐time on Archimed X4 qPCR (RocGene, Beijing) at 37°C, with the FAM channel. All experiments were performed with three independent replicates.

### 4.12 HOCl Detection

Hypochlorous acid solution (HClO solution) was generated by acidifying sodium hypochlorite with sulfuric acid. In a 5 µL reaction mixture, HClO solution at various concentration was mixed with 100 nM PT‐modified dsDNA activator, and then incubated at 37°C for 2 h. A 15 µL reaction mixture (1× Buffer r2.1, 25 nM scaffold RNA, 0.5 nM spacer RNA, 250 nM ssDNA reporter, 25 nM Cas12a) was added to the mixture. The fluorescence intensity of the reaction solution was monitored in real‐time on Archimed X4 qPCR (RocGene, Beijing) at 37°C, with the FAM channel.

To calculate the test recovery rate, the lake water sample was spiked with HClO to final concentrations of 25, 100, and 250 nM, while the swimming pool water sample was spiked to final concentrations of 250, 1000, and 2500 nM. In a 5 µL reaction mixture, 100 nM modified activator and water sample with various concentrations of HClO were mixed well, and incubated at 37°C for 2 h. Next, a 15 µL reaction mixture was added to the mixture. The fluorescence intensity of the reaction solution was monitored in real‐time on Archimed X4 qPCR (RocGene, Beijing) at 37°C, with the FAM channel. All experiments were performed with three independent replicates. The recovery rate for each group was calculated by comparing the measured fluorescence signal to the standard curve.

## Author Contributions

X.T. and J.Z. conceived and designed the study. Z.L. provided computational algorithm support. X.T., Y.L., M.M., Z.M., J.K., and X.L. carried out the experimental work and data analysis. J.Z. and T.L. supervised the project, secured funding, and offered conceptual direction. X.T. and J.Z. wrote the manuscript with input from all co‐authors, who reviewed and approved the final version.

## Conflicts of Interest

The authors declare no competing interests.

## Supporting information




**Supporting File**: advs76576‐sup‐0001‐SuppMat.doc.

## Data Availability

All the data supporting the findings of this study are available within the article and Supplementary Files. Source data are provided with this paper, and the code is available on GitHub.
