# Supplementary material for: Systematic Position Mapping of Split CRISPR‐Cas12a Activators Enables Highly Sensitive miRNA Detection and Cancer Cell Stratification
Source: Adv Sci (Weinh). 2026 Jul 23:e76576. Online ahead of print. doi: 10.1002/advs.76576 (PMC13393258; doi:10.1002/advs.76576)
Supplement: Supplementary file 1 — Supporting File: advs76576‐sup‐0001‐SuppMat.doc. [file ADVS-9999-e76576-s001.doc]

**Supplementary Information**

# Systematic position mapping of split CRISPR-Cas12a activators enables highly sensitive miRNA detection and cancer cell stratification

Xiaoyan Tang^1^, Zhe Li^2^, Yuning Lu^1^, Miao Ma^4^, Zijian Mo^1^, Jiajun Ke^1^, Xinyu Luan^4^, Tiangang Luan^3,4,*^, Junqiu Zhai^1,4,*^

^1^Key Laboratory of Chinese Medicinal Resource from Lingnan, Ministry of Education, School of Pharmaceutical Sciences, Guangzhou University of Chinese Medicine, Guangzhou, 510006, P. R. China

^2^Guangdong Provincial Key Laboratory of New Drug Design and Evaluation, School of Pharmaceutical Sciences, Sun Yat-Sen University, 510006 Guangzhou, China

^3^School of Environmental and Chemical Engineering, Wuyi University, Jiangmen 529020, China

^4^State Key Laboratory of Biocontrol, School of Life Sciences, Sun Yat-sen University, Guangzhou, 510275, China

**Oligonucleotide sequences used in this study**

**Table S1.** Sequences of split activators used in this work.

| **Name** | **Sequence (from 5’ to 3’)** |
| --- | --- |
| Pp-TS(0) | TAAAGGGCCATA |
| Pd-TS(20) | TCCAAGCTCAACACTGTACTGGAAGATG |
| Pp-TS(1) | GTAAAGGGCCATA |
| Pd-TS(19) | TCCAAGCTCAACACTGTACTGGAAGAT |
| Pp-TS(2) | TGTAAAGGGCCATA |
| Pd-TS(18) | TCCAAGCTCAACACTGTACTGGAAGA |
| Pp-TS(3) | ATGTAAAGGGCCATA |
| Pd-TS(17) | TCCAAGCTCAACACTGTACTGGAAG |
| Pp-TS(4) | GATGTAAAGGGCCATA |
| Pd-TS(16) | TCCAAGCTCAACACTGTACTGGAA |
| Pp-TS(5) | AGATGTAAAGGGCCATA |
| Pd-TS(15) | TCCAAGCTCAACACTGTACTGGA |
| Pp-TS(6) | AAGATGTAAAGGGCCATA |
| Pd-TS(14) | TCCAAGCTCAACACTGTACTGG |
| Pp-TS(7) | GAAGATGTAAAGGGCCATA |
| Pd-TS(13) | TCCAAGCTCAACACTGTACTG |
| Pp-TS(8) | GGAAGATGTAAAGGGCCATA |
| Pd-TS(12) | TCCAAGCTCAACACTGTACT |
| Pp-TS(9) | TGGAAGATGTAAAGGGCCATA |
| Pd-TS(11) | TCCAAGCTCAACACTGTAC |
| Pp-TS(10) | CTGGAAGATGTAAAGGGCCATA |
| Pd-TS(10) | TCCAAGCTCAACACTGTA |
| Pp-TS(11) | ACTGGAAGATGTAAAGGGCCATA |
| Pd-TS(9) | TCCAAGCTCAACACTGT |
| Pp-TS(12) | TACTGGAAGATGTAAAGGGCCATA |
| Pd-TS(8) | TCCAAGCTCAACACTG |
| Pp-TS(13) | GTACTGGAAGATGTAAAGGGCCATA |
| Pd-TS(7) | TCCAAGCTCAACACT |
| Pp-TS(14) | TGTACTGGAAGATGTAAAGGGCCATA |
| Pd-TS(6) | TCCAAGCTCAACAC |
| Pp-TS(15) | CTGTACTGGAAGATGTAAAGGGCCATA |
| Pd-TS(5) | TCCAAGCTCAACA |
| Pp-TS(16) | ACTGTACTGGAAGATGTAAAGGGCCATA |
| Pd-TS(4) | TCCAAGCTCAAC |
| Pp-TS(17) | CACTGTACTGGAAGATGTAAAGGGCCATA |
| Pd-TS(3) | TCCAAGCTCAA |
| Pp-TS(18) | ACACTGTACTGGAAGATGTAAAGGGCCATA |
| Pd-TS(2) | TCCAAGCTCA |
| Pp-TS(19) | AACACTGTACTGGAAGATGTAAAGGGCCATA |
| Pd-TS(1) | TCCAAGCTC |
| Pp-TS(20) | CAACACTGTACTGGAAGATGTAAAGGGCCATA |
| Pd-TS(0) | TCCAAGCT |
| Pp-NTS(0) | TATGGCCCTTTA |
| Pd-NTS(20) | CATCTTCCAGTACAGTGTTGAGCTTGGA |
| Pp-NTS(1) | TATGGCCCTTTAC |
| Pd-NTS(19) | ATCTTCCAGTACAGTGTTGAGCTTGGA |
| Pp-NTS(2) | TATGGCCCTTTACA |
| Pd-NTS(18) | TCTTCCAGTACAGTGTTGAGCTTGGA |
| Pp-NTS(3) | TATGGCCCTTTACAT |
| Pd-NTS(17) | CTTCCAGTACAGTGTTGAGCTTGGA |
| Pp-NTS(4) | TATGGCCCTTTACATC |
| Pd-NTS(16) | TTCCAGTACAGTGTTGAGCTTGGA |
| Pp-NTS(5) | TATGGCCCTTTACATCT |
| Pd-NTS(15) | TCCAGTACAGTGTTGAGCTTGGA |
| Pp-NTS(6) | TATGGCCCTTTACATCTT |
| Pd-NTS(14) | CCAGTACAGTGTTGAGCTTGGA |
| Pp-NTS(7) | TATGGCCCTTTACATCTTC |
| Pd-NTS(13) | CAGTACAGTGTTGAGCTTGGA |
| Pp-NTS(8) | TATGGCCCTTTACATCTTCC |
| Pd-NTS(12) | AGTACAGTGTTGAGCTTGGA |
| Pp-NTS(9) | TATGGCCCTTTACATCTTCCA |
| Pd-NTS(11) | GTACAGTGTTGAGCTTGGA |
| Pp-NTS(10) | TATGGCCCTTTACATCTTCCAG |
| Pd-NTS(10) | TACAGTGTTGAGCTTGGA |
| Pp-NTS(11) | TATGGCCCTTTACATCTTCCAGT |
| Pd-NTS(9) | ACAGTGTTGAGCTTGGA |
| Pp-NTS(12) | TATGGCCCTTTACATCTTCCAGTA |
| Pd-NTS(8) | CAGTGTTGAGCTTGGA |
| Pp-NTS(13) | TATGGCCCTTTACATCTTCCAGTAC |
| Pd-NTS(7) | AGTGTTGAGCTTGGA |
| Pp-NTS(14) | TATGGCCCTTTACATCTTCCAGTACA |
| Pd-NTS(6) | GTGTTGAGCTTGGA |
| Pp-NTS(15) | TATGGCCCTTTACATCTTCCAGTACAG |
| Pd-NTS(5) | TGTTGAGCTTGGA |
| Pp-NTS(16) | TATGGCCCTTTACATCTTCCAGTACAGT |
| Pd-NTS(4) | GTTGAGCTTGGA |
| Pp-NTS(17) | TATGGCCCTTTACATCTTCCAGTACAGTG |
| Pd-NTS(3) | TTGAGCTTGGA |
| Pp-NTS(18) | TATGGCCCTTTACATCTTCCAGTACAGTGT |
| Pd-NTS(2) | TGAGCTTGGAA |
| Pp-NTS(19) | TATGGCCCTTTACATCTTCCAGTACAGTGTT |
| Pd-NTS(1) | GAGCTTGGAAA |
| Pp-NTS(20) | TATGGCCCTTTACATCTTCCAGTACAGTGTTG |
| Pd-NTS(0) | AGCTTGGAAAA |
| full-TS | TCCAAGCTCAACACTGTACTGGAAGATGTAAAGGGCCATA |
| full-NTS | TATGGCCCTTTACATCTTCCAGTACAGTGTTGAGCTTGGA |
| Pd-TS(20)-ROX | /ROX/TCCAAGCTCAACACTGTACTGGAAGATG |
| Pd-TS(15)-ROX | /ROX/TCCAAGCTCAACACTGTACTGGA |
| Pd-TS(10)-ROX | /ROX/TCCAAGCTCAACACTGTA |
| Pd-TS(5)-ROX | /ROX/TCCAAGCTCAACA |
| Pd-TS(0)-ROX | /ROX/TCCAAGCT |
| full-TS-ROX | /ROX/TCCAAGCTCAACACTGTACTGGAAGATGTAAAGGGCCATA |
| Pd-NTS(20)-BHQ2 | CATCTTCCAGTACAGTGTTGAGCTTGGA/BHQ2/ |
| Pd-NTS(15)-BHQ2 | TCCAGTACAGTGTTGAGCTTGGA/BHQ2/ |
| Pd-NTS(10)-BHQ2 | TACAGTGTTGAGCTTGGA/BHQ2/ |
| Pd-NTS(5)-BHQ2 | TGTTGAGCTTGGA/BHQ2/ |
| Pd-NTS(0)-BHQ2 | AGCTTGGAAAA/BHQ2/ |
| full-NTS-BHQ2 | TATGGCCCTTTACATCTTCCAGTACAGTGTTGAGCTTGGA/BHQ2/ |
| scaffold RNA (DR) | UAAUUUCUACUAAGUUGUAGAU |
| spacer RNA | CAUCUUCCAGUACAGUGUUGGA |
| spacer RNA-BHQ2 | CAUCUUCCAGUACAGUGUUGGA/BHQ2/ |
| ssDNA reporter (F-Q) | /FAM/CCCCCCCC/BHQ1/ |

Each strand of split activator divides the activator sequence (pink) into two segments: the PAM (yellow) -proximal segment (Pp) and PAM-distal (Pd) segment. The numbers in parentheses indicate the length (in bases) of the activated region in oligonucleotide.

**Table S2.** DNA sequences used for multi-molecule detection and RNA target in this work.

| **Name** | **Sequence (from 5’ to 3’)** |
| --- | --- |
| Pp-TS(11)-21 | CTGATAAGCTATAAAGGGCCATA |
| Pd-TS(9)-21 | TCCAAGCTAACATCAGT |
| Pp-NTS(5)-21 | TATGGCCCTTTATAGCT |
| Pd-NTS(15)-21 | TATCAGACTGATGTTAGCTTGGA |
| Pp-TS(11)-155 | ATTAGCATTAATAAAGGGCCATA |
| Pd-TS(9)-155 | TCCAAGCTCCTATCACG |
| Pp-NTS(5)-155 | TATGGCCCTTTATTAAT |
| Pd-NTS(15)-155 | GCTAATCGTGATAGGAGCTTGGA |
| Pp-TS(11)-429 | AGACAGTATTATAAAGGGCCATA |
| Pd-TS(9)-429 | TCCAAGCTGGTTTTACC |
| Pp-NTS(5)-429 | TATGGCCCTTTATAATA |
| Pd-NTS(15)-429 | CTGTCTGGTAAAACCAGCTTGGA |
| Pp-TS(11)-141 | ACTGGAAGATGTAAAGGGCCATA |
| Pd-TS(9)-141 | TCCAAGCTCAACACTGT |
| Pp-NTS(5)-141 | TATGGCCCTTTACATCT |
| Pd-NTS(15)-141 | TCCAGTACAGTGTTGAGCTTGGA |
| Pp-TS(11)-222 | TGGCTACTGAGTAAAGGGCCATA |
| Pd-TS(9)-222 | TCCAAGCTGATCTACAC |
| Pp-NTS(5)-222 | TATGGCCCTTTACTCAG |
| Pd-NTS(15)-222 | TAGCCAGTGTAGATCAGCTTGGA |
| Pp-TS(11)-221 | ACAATGTAGCTTAAAGGGCCATA |
| Pd-TS(9)-221 | TCCAAGCTACCCAGCAG |
| Pp-NTS(5)-221 | TATGGCCCTTTAAGCTA |
| Pd-NTS(15)-221 | CATTGTCTGCTGGGTAGCTTGGA |
| Pp-TS(11)-MS2 1 | GTACGCCTTCATAAAGGGCCATA |
| Pd-TS(9)-MS2 1 | TCCAAGCTAGCGGCAGT |
| Pp-NTS(5)-MS2 1 | TATGGCCCTTTATGAAG |
| Pd-NTS(15)-MS2 1 | GCGTACACTGCCGCTAGCTTGGA |
| Pp-TS(11)-MS2 26 | GCCAATTACCGTAAAGGGCCATA |
| Pd-TS(9)-MS2 26 | TCCAAGCTGCGCCTGGC |
| Pp-NTS(5)-MS2 26 | TATGGCCCTTTACGGTA |
| Pd-NTS(15)-MS2 26 | ATTGGCGCCAGGCGCAGCTTGGA |
| Pp-TS(11)-MS2 51 | AGGGCAAGGTATAAAGGGCCATA |
| Pd-TS(9)-MS2 51 | TCCAAGCTTCTTCGTTT |
| Pp-NTS(5)-MS2 51 | TATGGCCCTTTATACCT |
| Pd-NTS(15)-MS2 51 | TGCCCTAAACGAAGAAGCTTGGA |
| Pp-TS(11)-MS2 76 | TGATCGAAACTTAAAGGGCCATA |
| Pd-TS(9)-MS2 76 | TCCAAGCTCACGTGTTT |
| Pp-NTS(5)-MS2 76 | TATGGCCCTTTAAGTTT |
| Pd-NTS(15)-MS2 76 | CGATCAAAACACGTGAGCTTGGA |
| Pp-TS(11)-MS2 81 | TGTTTTGATCGTAAAGGGCCATA |
| Pd-TS(9)-MS2 81 | TCCAAGCTCCGGCCACG |
| Pp-NTS(5)-MS2 81 | TATGGCCCTTTACGATC |
| Pd-NTS(15)-MS2 81 | AAAACACGTGGCCGGAGCTTGGA |
| full-TS-MS2 1 | TCCAAGCTAGCGGCAGTGTACGCCTTCATAAAGGGCCATA |
| full-NTS-MS2 1 | TATGGCCCTTTATGAAGGCGTACACTGCCGCTAGCTTGGA |
| full-TS-MS2 26 | TCCAAGCTGCGCCTGGCGCCAATTACCGTAAAGGGCCATA |
| full-NTS-MS2 26 | TATGGCCCTTTACGGTAATTGGCGCCAGGCGCAGCTTGGA |
| full-TS-MS2 51 | TCCAAGCTTCTTCGTTTAGGGCAAGGTATAAAGGGCCATA |
| full-NTS-MS2 51 | TATGGCCCTTTATACCTTGCCCTAAACGAAGAAGCTTGGA |
| full-TS-MS2 81 | TCCAAGCTCCGGCCACGTGTTTTGATCGTAAAGGGCCATA |
| full-NTS-MS2 81 | TATGGCCCTTTACGATCAAAACACGTGGCCGGAGCTTGGA |
| PT-NTS | TATGGCCCTTTACATCT*TCCAGTACAGTGTTGAGCTTGGA |
| AP-NTS | TATGGCCCTTTACATCT/idSp/CCAGTACAGTGTTGAGCTTGGA |
| miR-141 | CAUCUUCCAGUACAGUGUUGGA |
| miR-155 | UUAAUGCUAAUCGUGAUAGGGGU |
| miR-429 | UAAUACUGUCUGGUAAAACCGU |
| miR-222 | CUCAGUAGCCAGUGUAGAUCCU |
| miR-21 | UAGCUUAUCAGACUGAUGUUGA |
| miR-221 | AGCUACAUUGUCUGCUGGGUUUC |
| MS2 RNA | UGAAGGCGUACACUGCCGCUCUCGCGGUAAUUGGCGCCAGGCGCUCCGCUACCUUGCCCUAAACGAAGAUCGAAAGUUUCGAUCAAAACACGUGGCCGG |

* represent the phosphorothioate modifications in the DNA backbone.

**Supporting Figures**


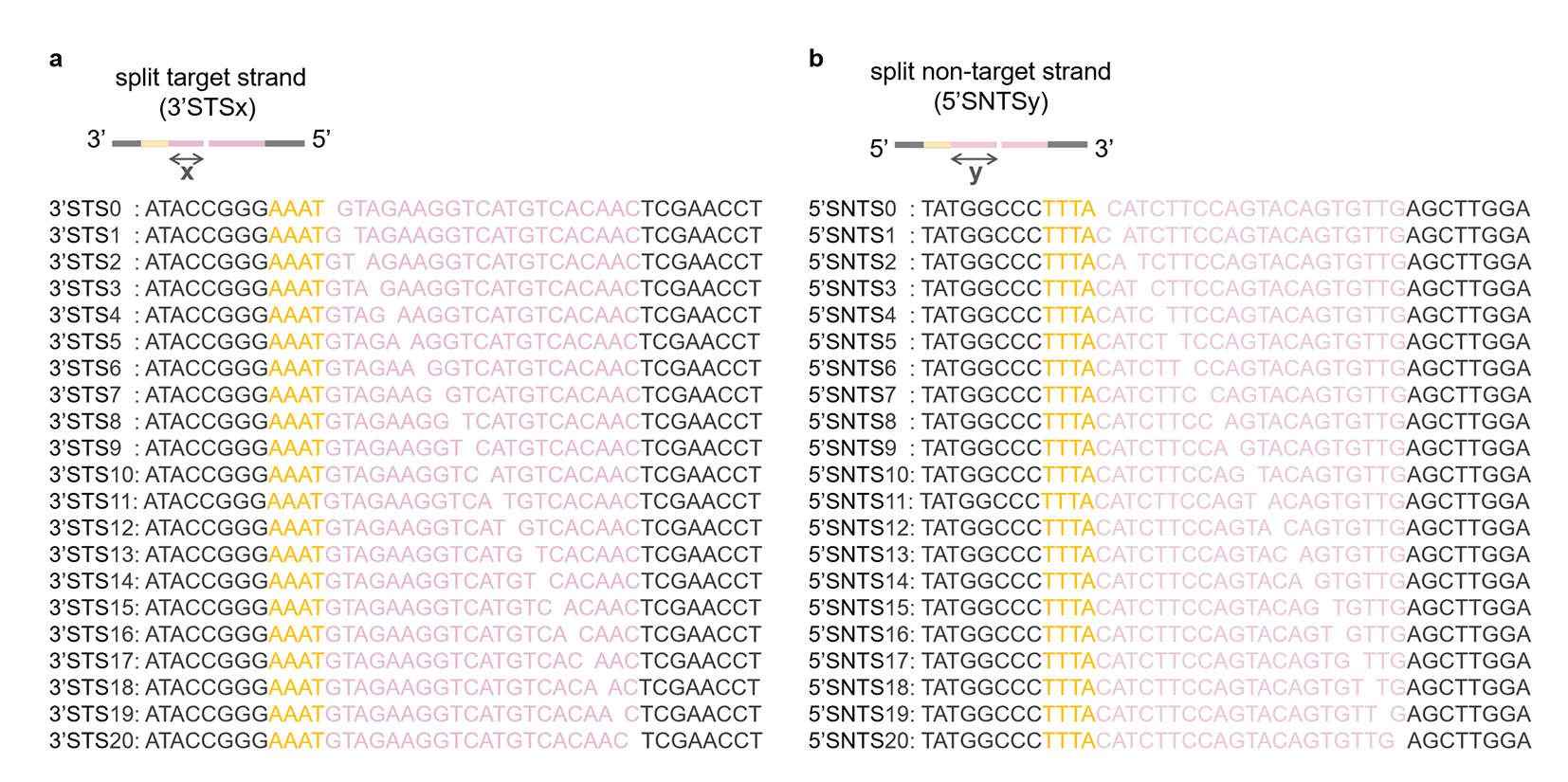


**Figure S1.** Design of breaks in **a** target strand and **b** non-target strand. ‘x’ and ‘y’ indicate the positions of nick site after the x th and y th bases, respectively.


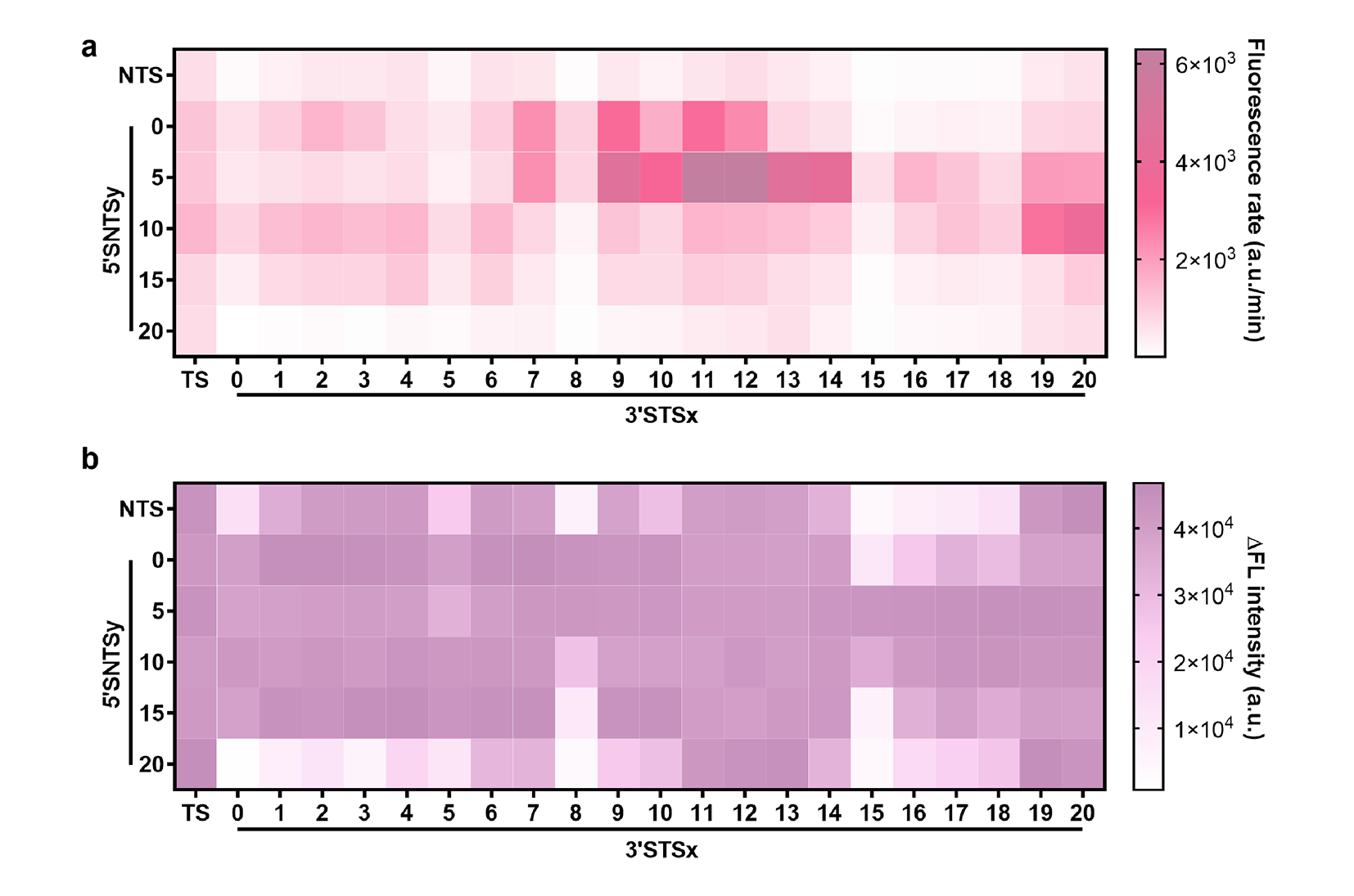


**Figure S2.** Systematic investigation of the effects of nicks located in TS on Cas12a. **a** Fluorescence rates and **b** signal increases of *trans*-cleavage activity at each nick site in the TS paired with NTS, 5’SNTS0, 5’SNTS5, 5’SNTS10, 5’SNTS15, and 5’SNTS20. ΔFL intensity = FL_120 min_ – FL_0 min_. Reactions were incubated for 120 min at 37℃. The data represents mean of three technical replicates.


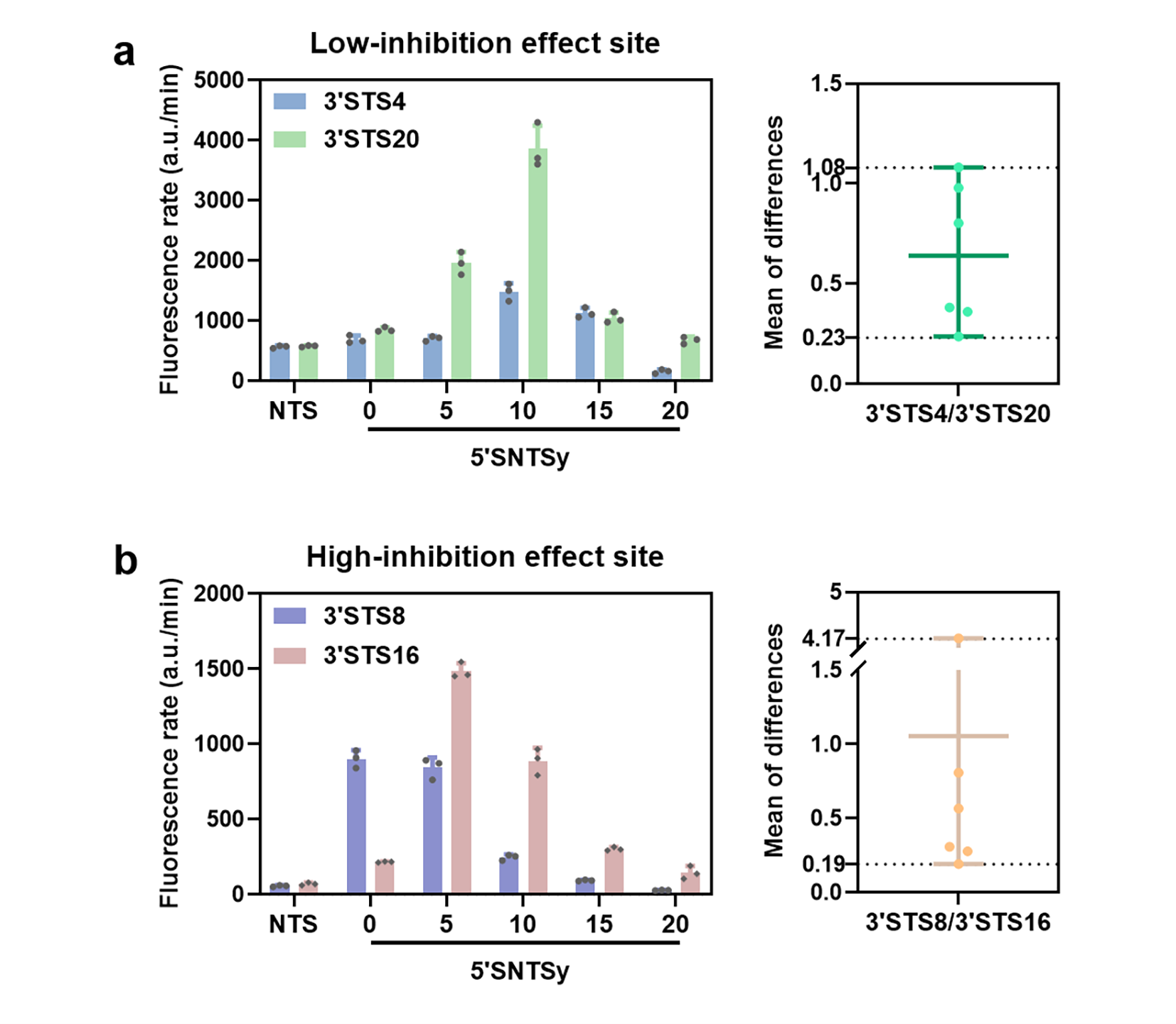


**Figure S3.** TS nick sites with comparable inhibitory effects exhibit differential alleviation by the same nicked NTS. **a** *Trans*-cleavage rates comparison of two split TS with low inhibition across various nicked NTS. **b** Comparison of two split TS with high inhibition under various nicked NTS. Reactions were incubated for 120 min at 37℃. The data represent mean of three technical replicates.


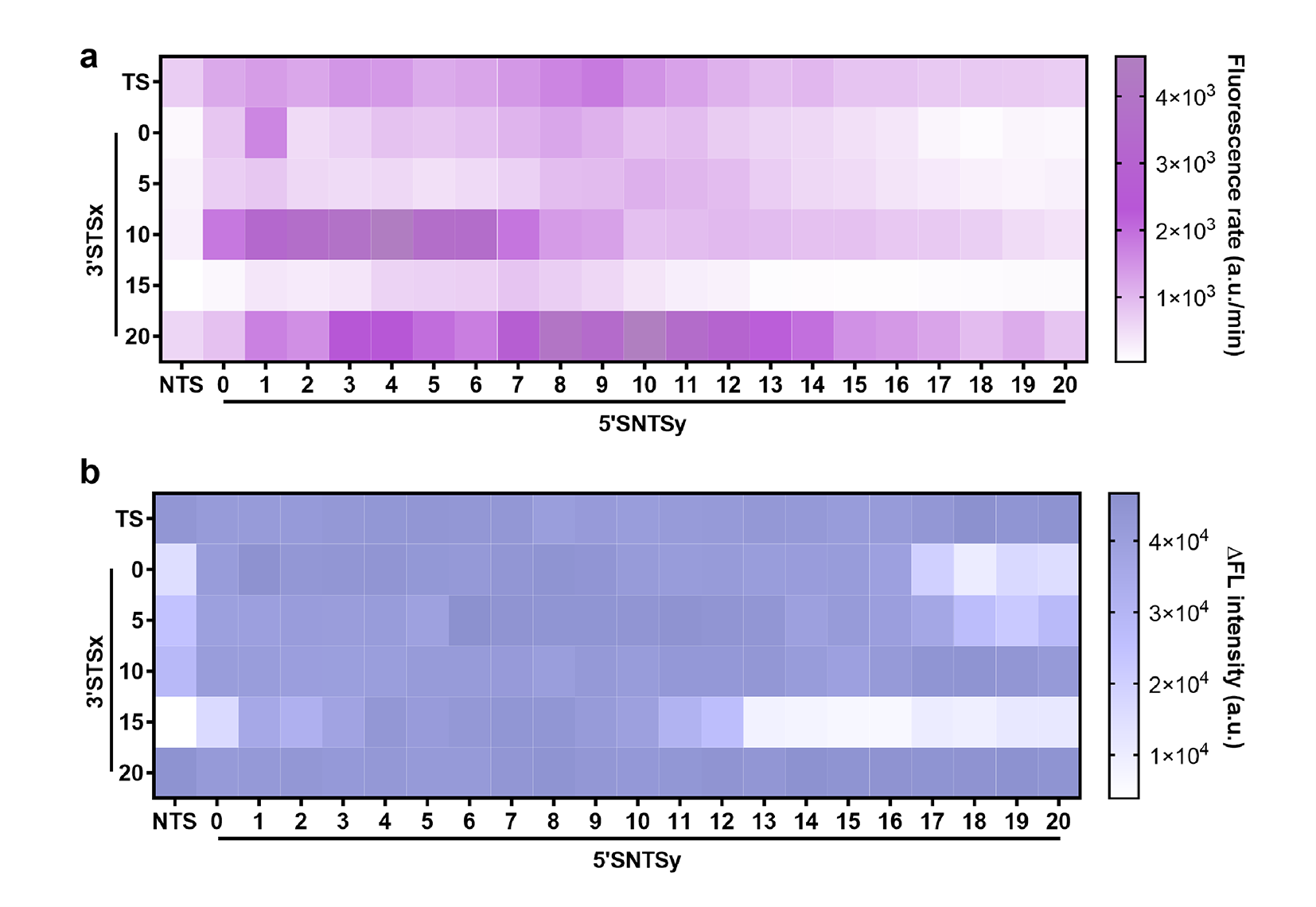


**Figure S4.** Matrix of *trans*-cleavage activity at each nick site in the NTS paired with TS and various nicked TS. ΔFL intensity = FL_120 min_ – FL_0 min_. Reactions were incubated for 120 min at 37℃. The data represents mean of three technical replicates.


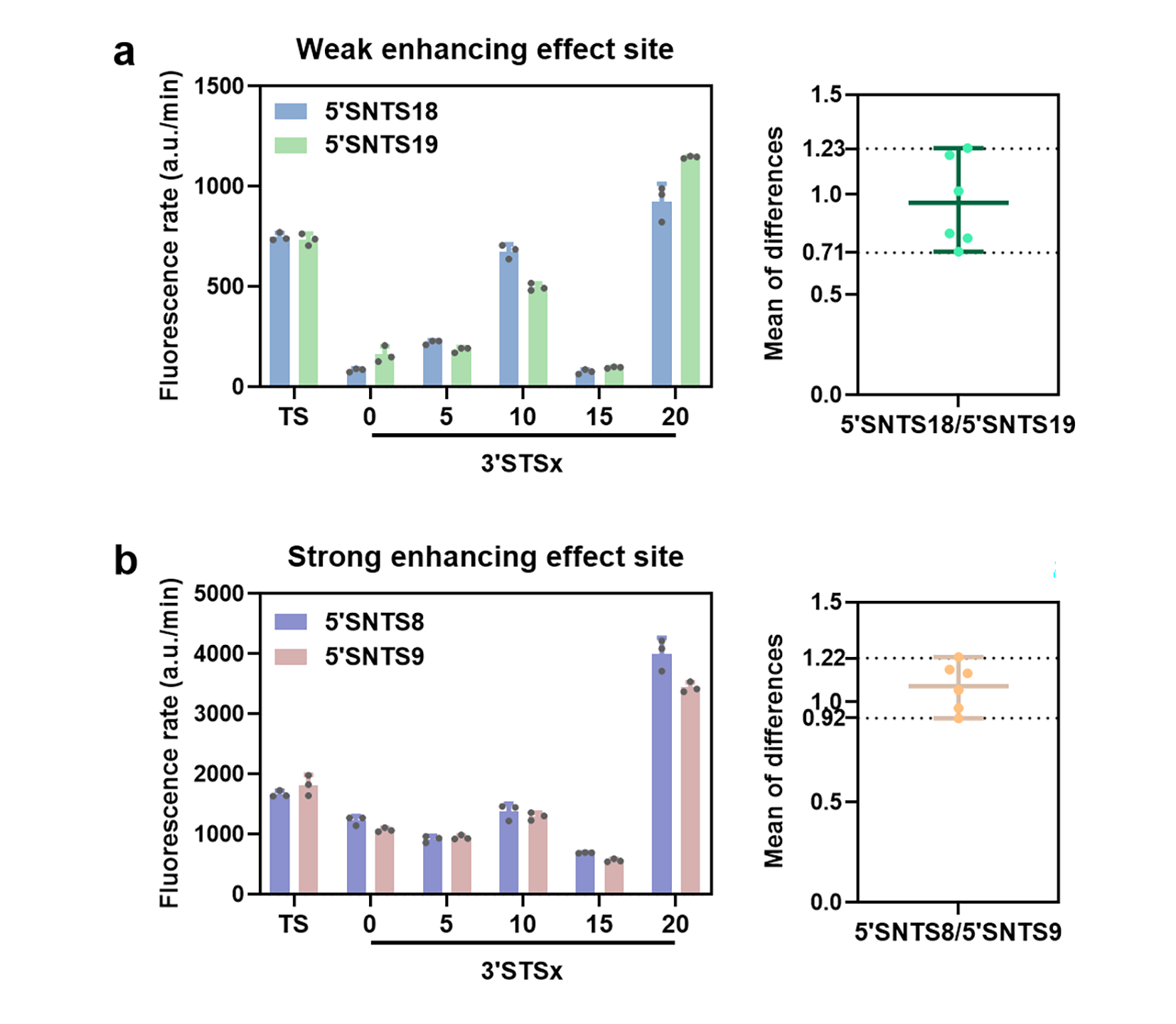


**Figure S5.** NTS nick sites with comparable promoting effects exhibit similar suppression when paired with the same nicked TS. **a** *Trans*-cleavage rates comparison of two split NTS with weak enhancing across various nicked TS. **b** Comparison of two split TS with strong enhancing under various nicked NTS. Reactions were incubated for 120 min at 37℃. The data represent mean of three technical replicates.


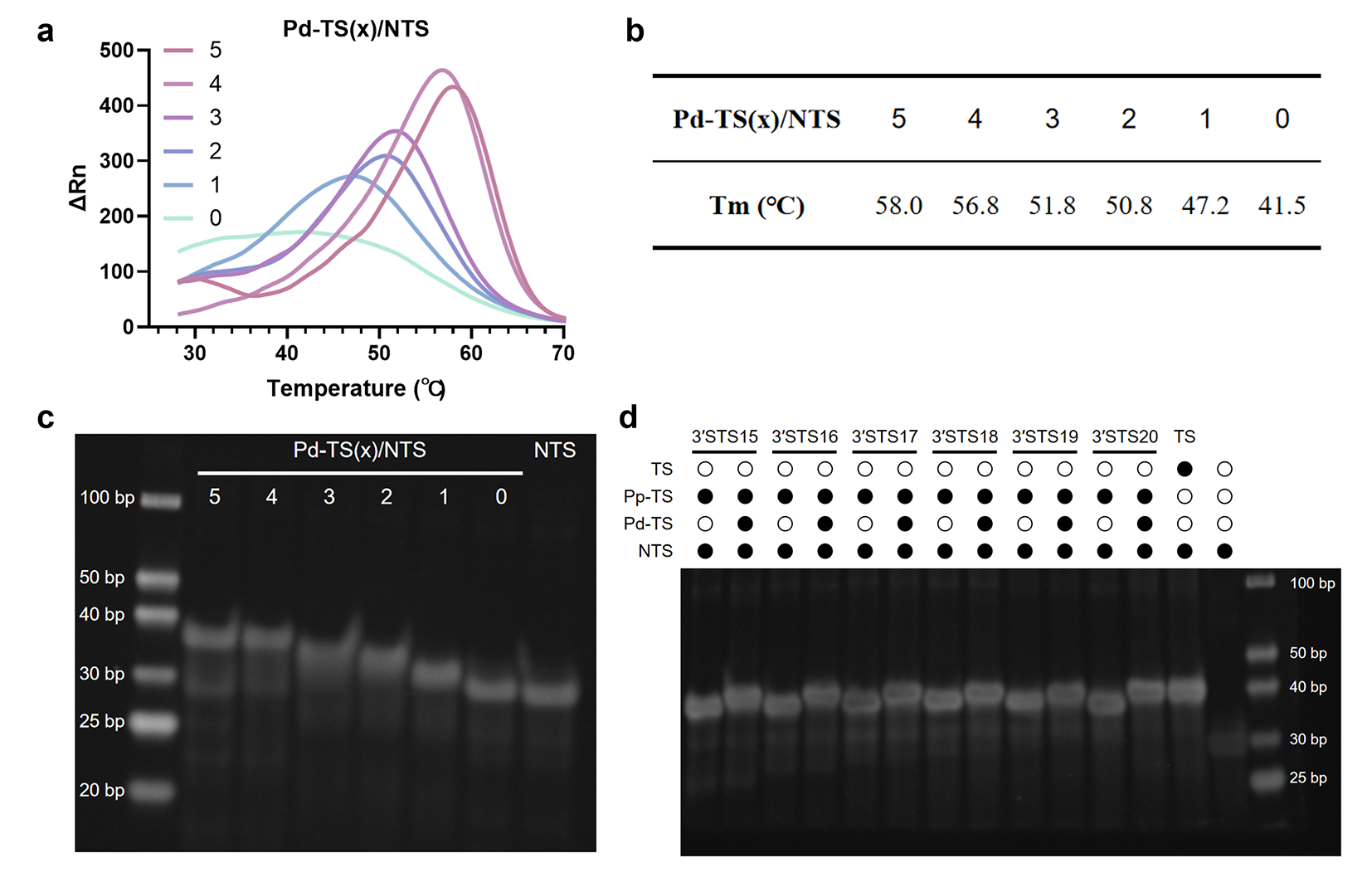


**Figure S6.** **a-b** The melting curves and values of the Pd-TS(x)/NTS complexs. **c** Native-PAGE analysis of Pd-TS(x)/NTS complexs. **d** Native-PAGE assay to evaluate triplex complex formation among Pd-TS, Pp-TS, and NTS strands.


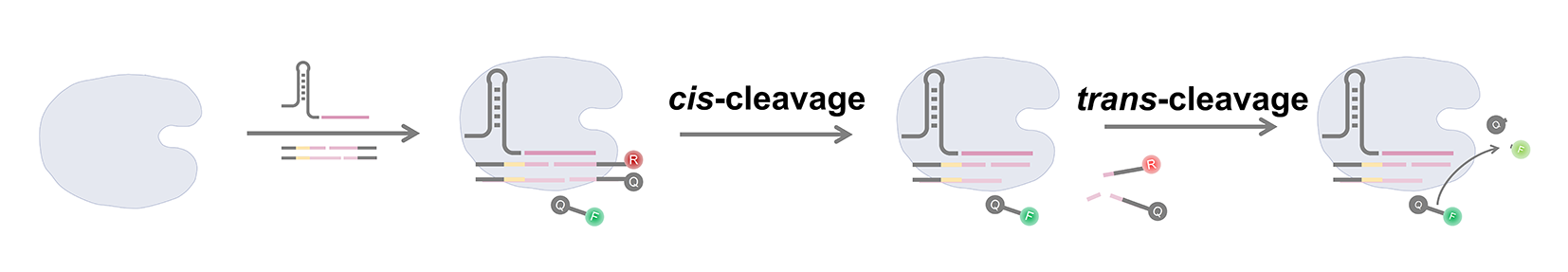


**Figure S7.** Illustration of Cas12a activation process.


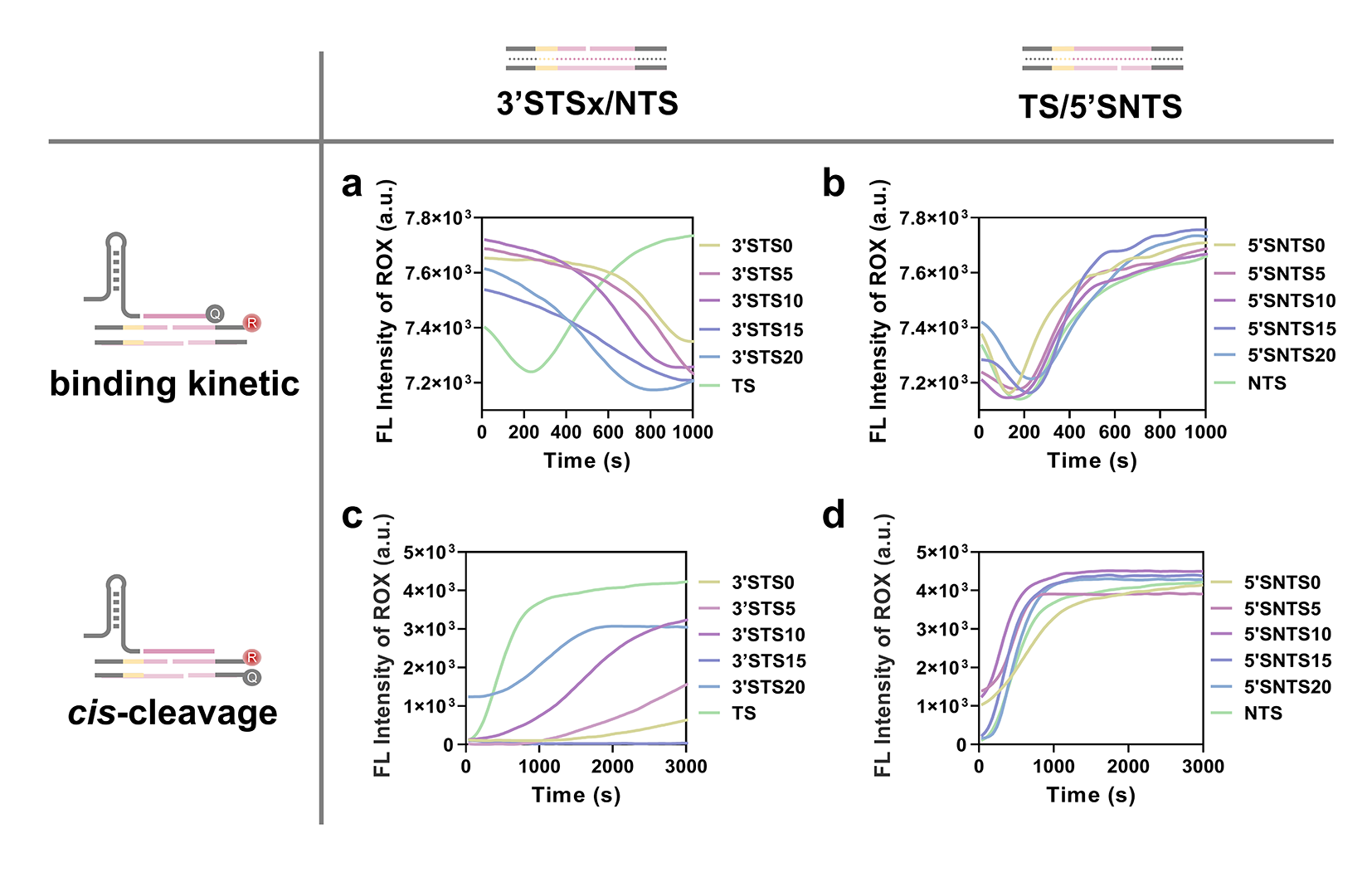


**Figure S8.** **a-b** The fluorescence curve for the split activator-crRNA binding. **c-d** The *cis*-cleavage activity curves of **c** 3’STSx/NTS and **d** TS/5’SNTSy.


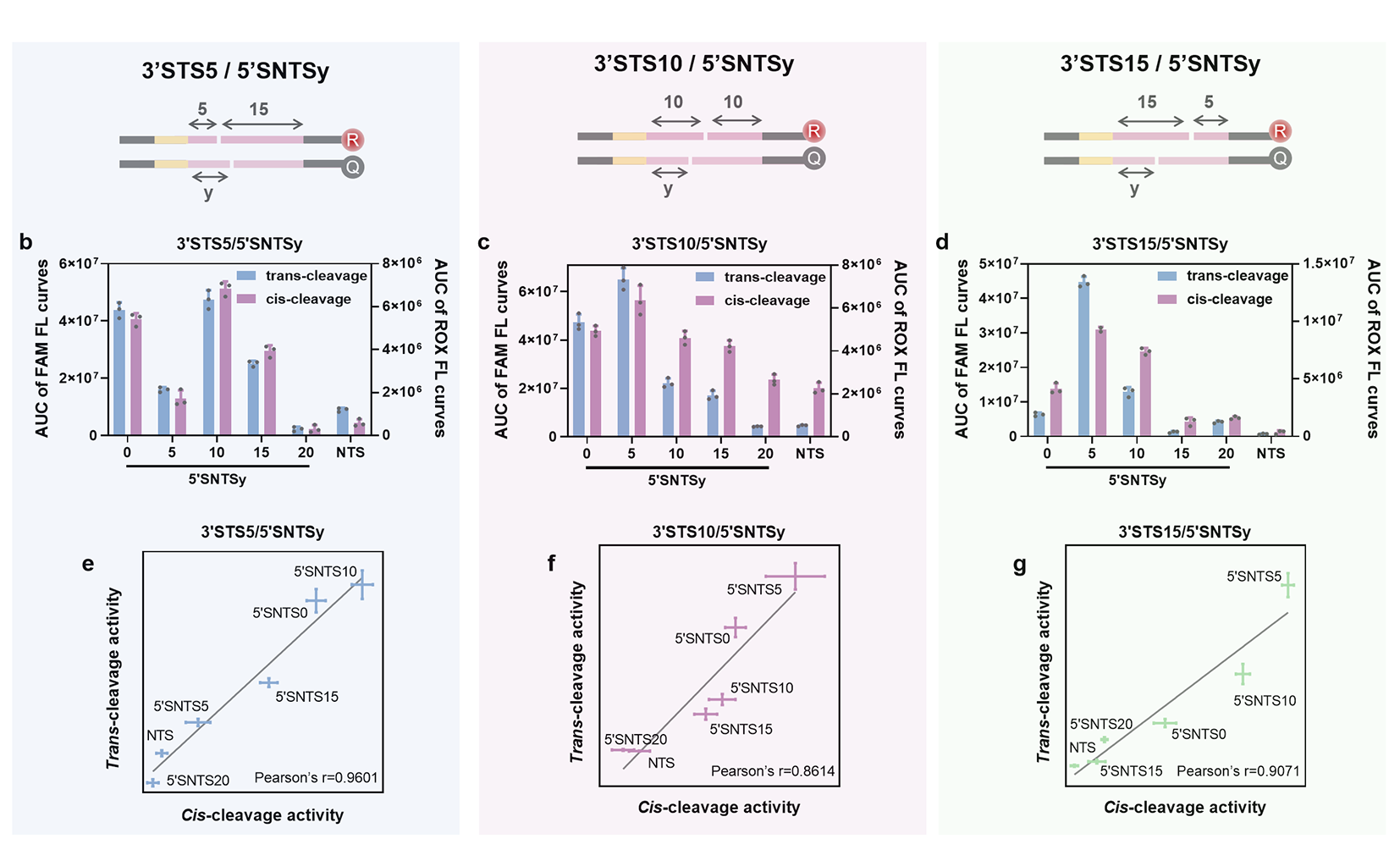


**Figure S9.** Comparson of *cis*-cleavage and *trans*-cleavage activity across combinations of internal nicks in the activator. **a**-**c** Histogram analysis of *cis*-cleavage and *trans*-cleavage activity. **e**-**g** Linear correlation between *cis*- and *trans*-cleavage activities. The data represent mean ± s.d. of three technical replicates.


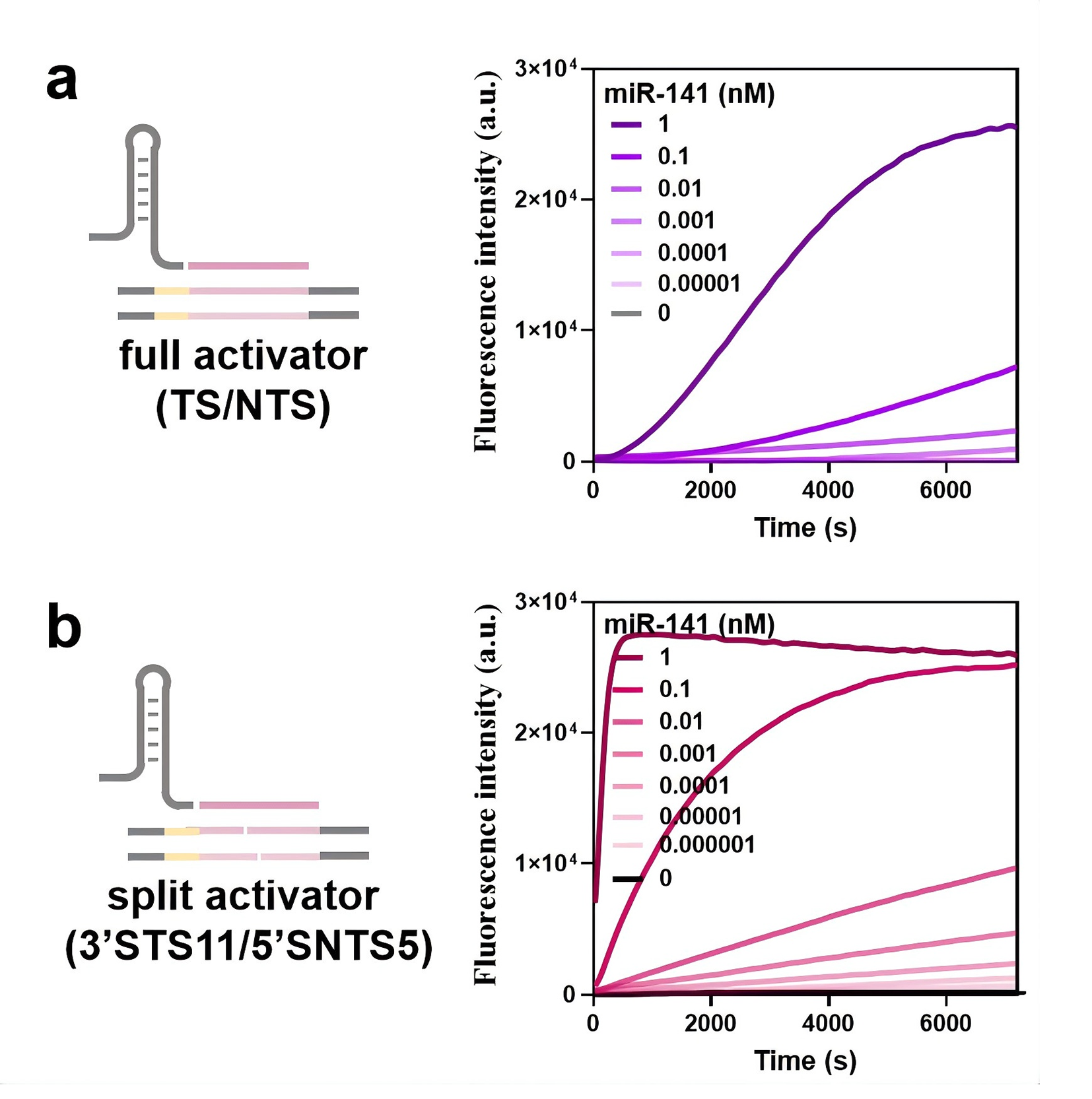


**Figure S10.** Time-dependent fluorescence signal of **a** split and **b** full dsDNA activators as a function of miR-141 concentrations.


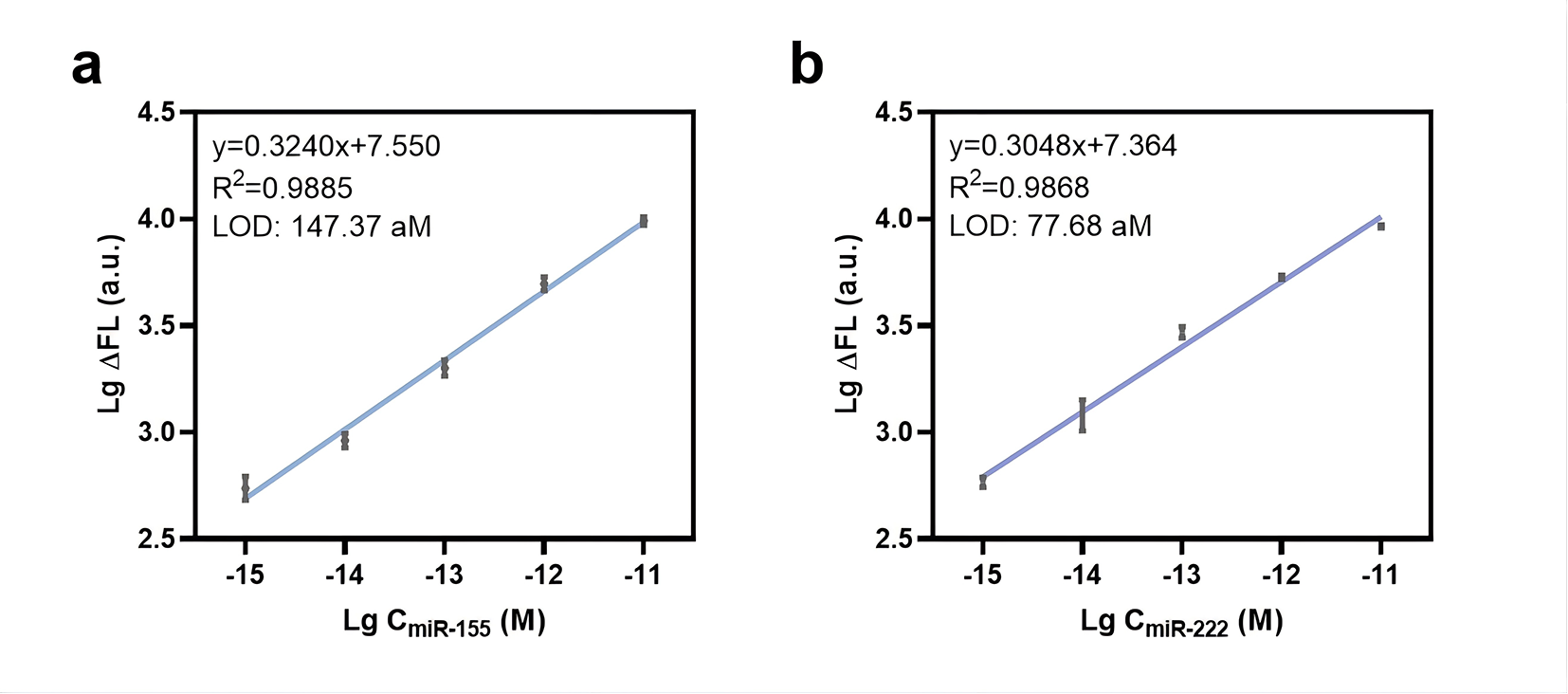


**Figure S11.** The linear relationship between the logarithm of fluorescence enhancement and the logarithm of concentration of **a** miR-155 and **b** miR-222.


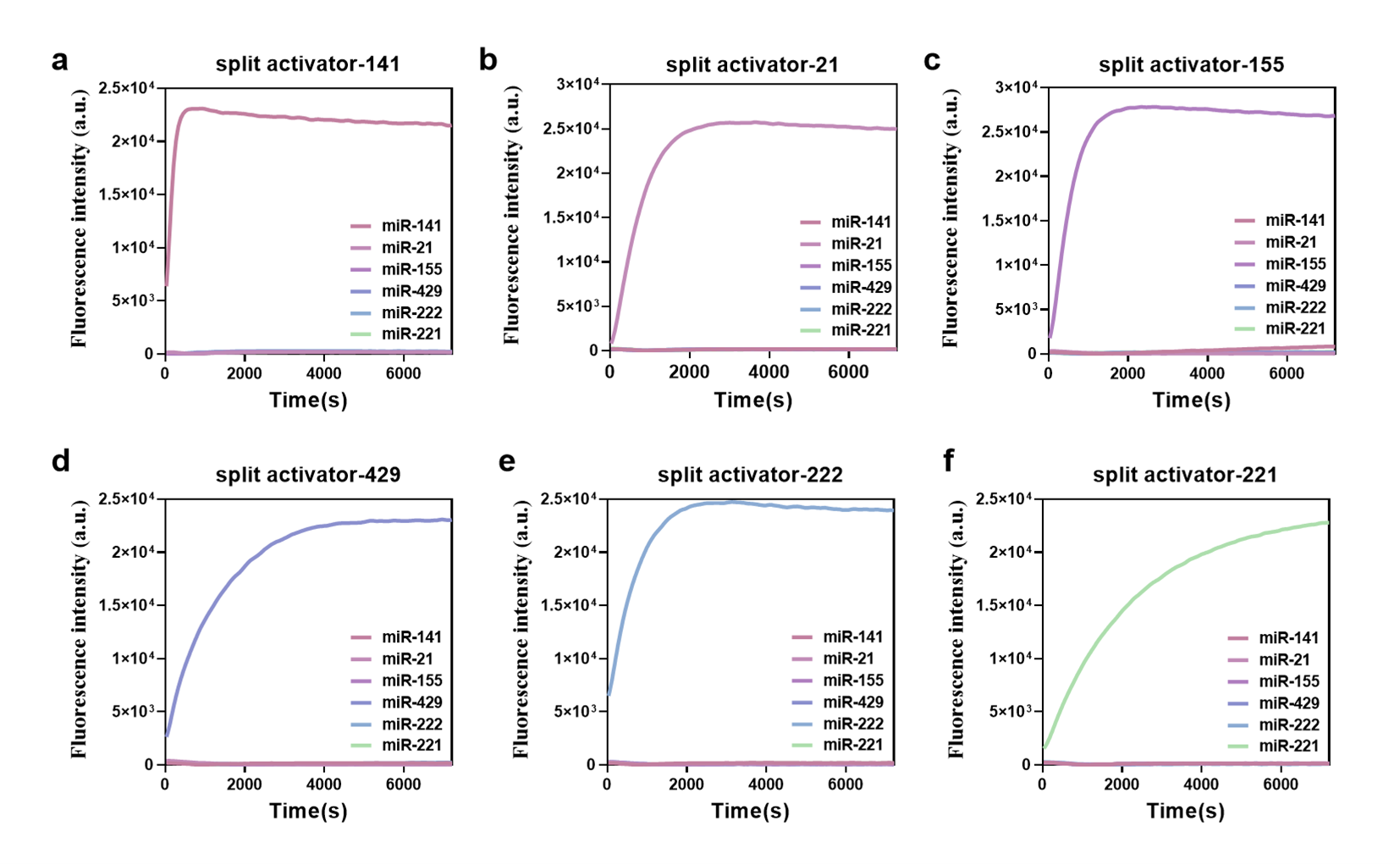


**Figure S12.** Time-dependent fluorescence signal changes of *trans*-cleavage activity for different miRNAs tested with split activator- **a** 141, **b** 21, **c** 155, **d** 429, **e** 222, and **f** 221.


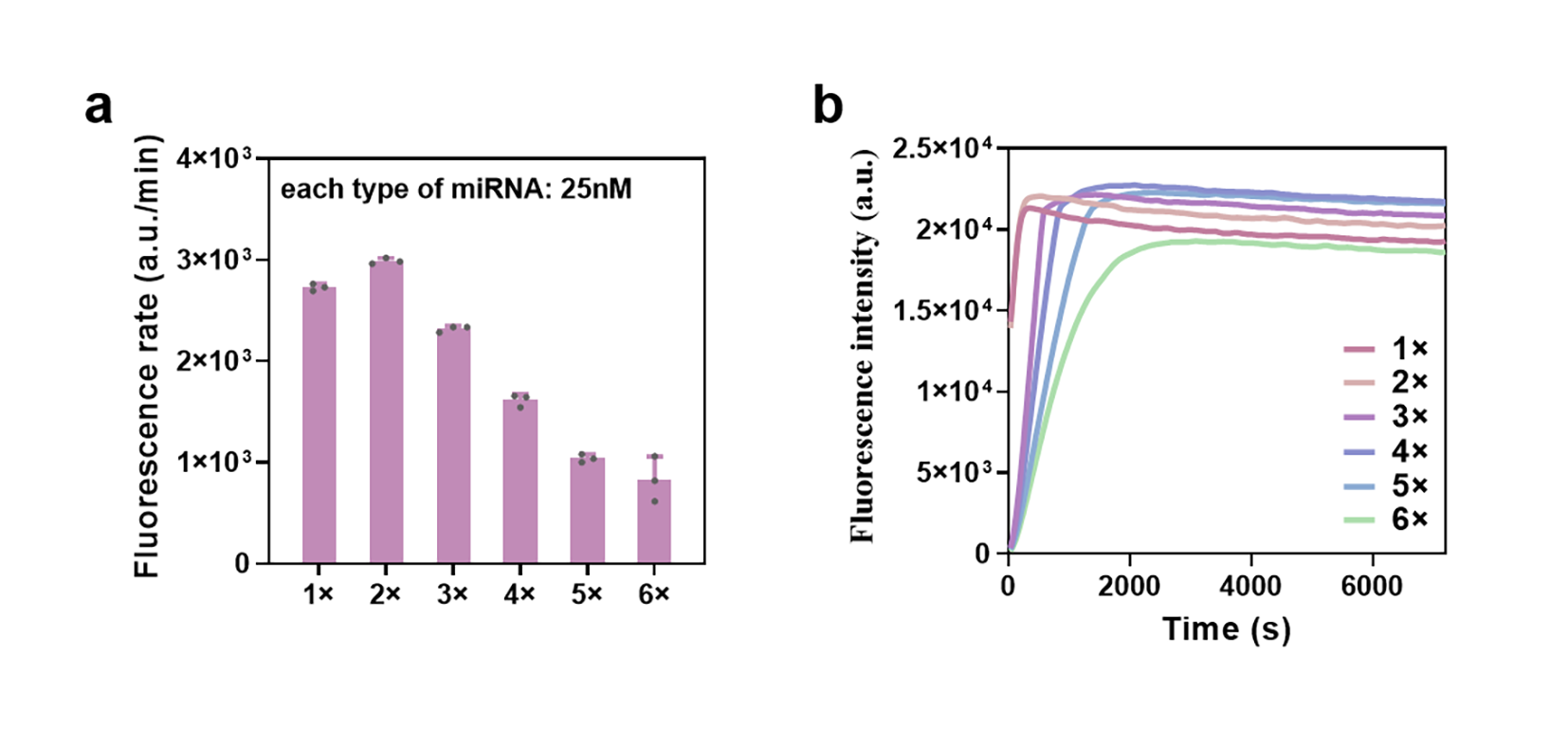


**Figure S13.** **a** Fluorescence generation rate and **b** kinetics curves of target miRNA mixed with various types of random miRNA, each at 25nM. The data represent mean ± s.d. of three technical replicates.


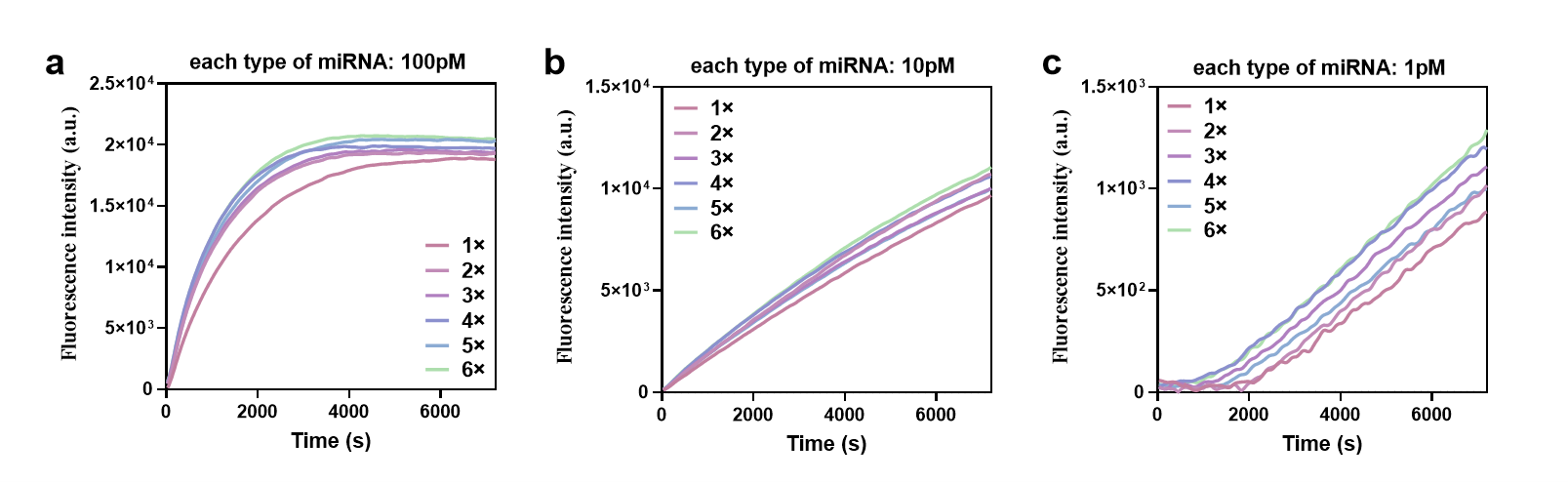


**Figure S14.** Time-dependent fluorescence increase curves of target miRNA mixed with various types of random miRNA, each at **a** 100 pM, **b** 10 pM, or **c** 1 pM.


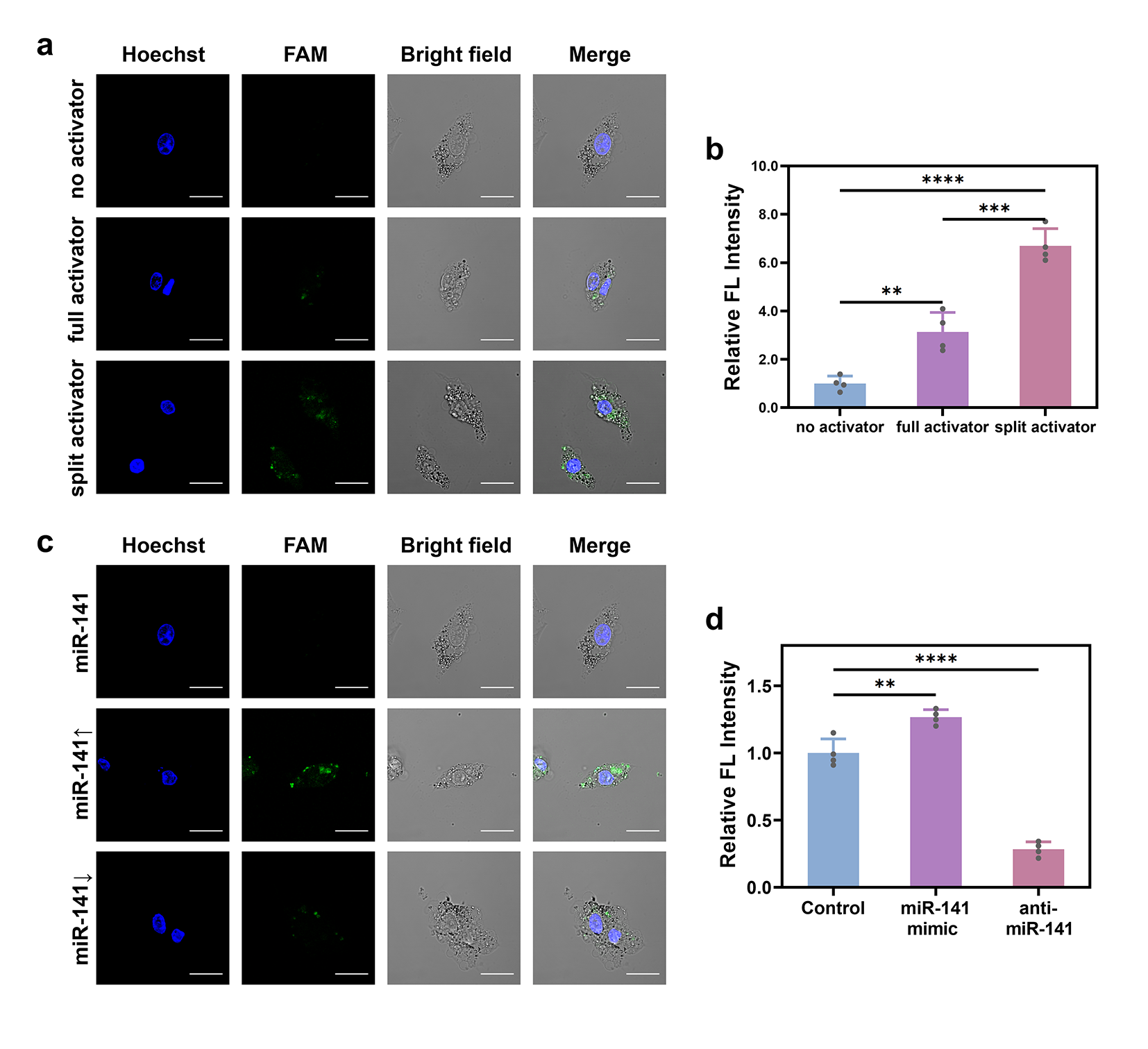


**Figure S15.** Analysis of miR-141 in HepG2 cells. **a** Confocal fluorescence images and **b** relative fluorescence intensity of miR-141 detection in HepG2 cells incubated with split crRNA-guided Cas12a system. **c** Confocal fluorescence images and **d** relative fluorescence intensity of HepG2 cells pretreated with miR-141 mimics or anti-miR-141, using the Cas12a system activated by split activator. Scale bars=20 μm. The data represent mean ± S.D of four technical replicates.


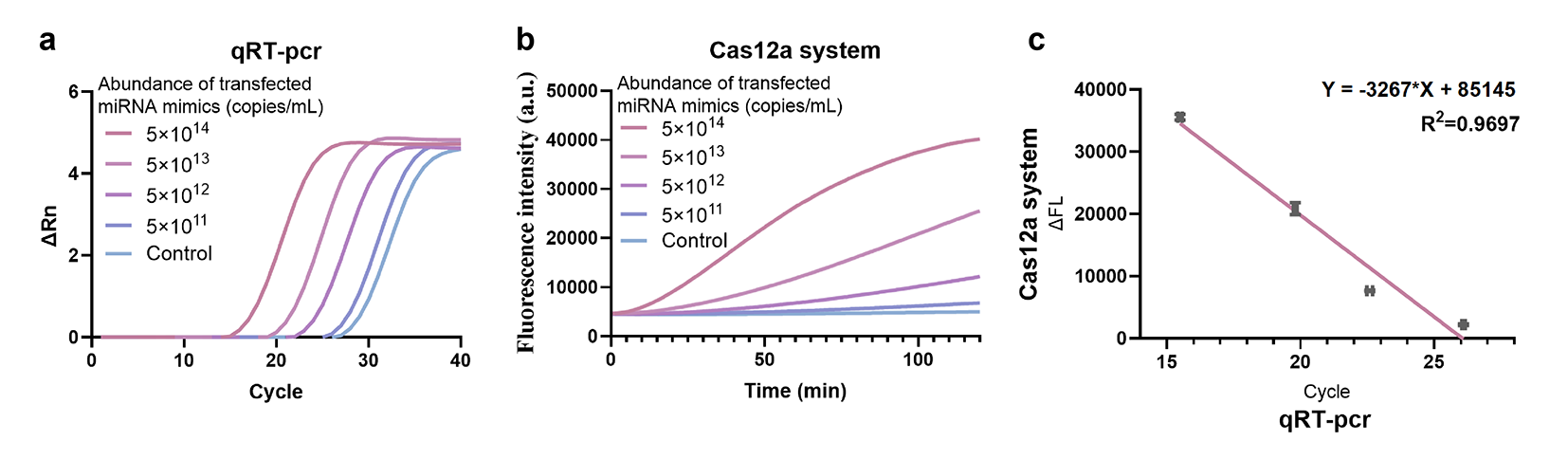


**Figure S16.** **a** The qRT-PCR analysis and **b** Cas12a system detection of miRNA abundance in cell lysates. **c** Linear correlation between the two methods (qRT-PCR and Cas12a system).


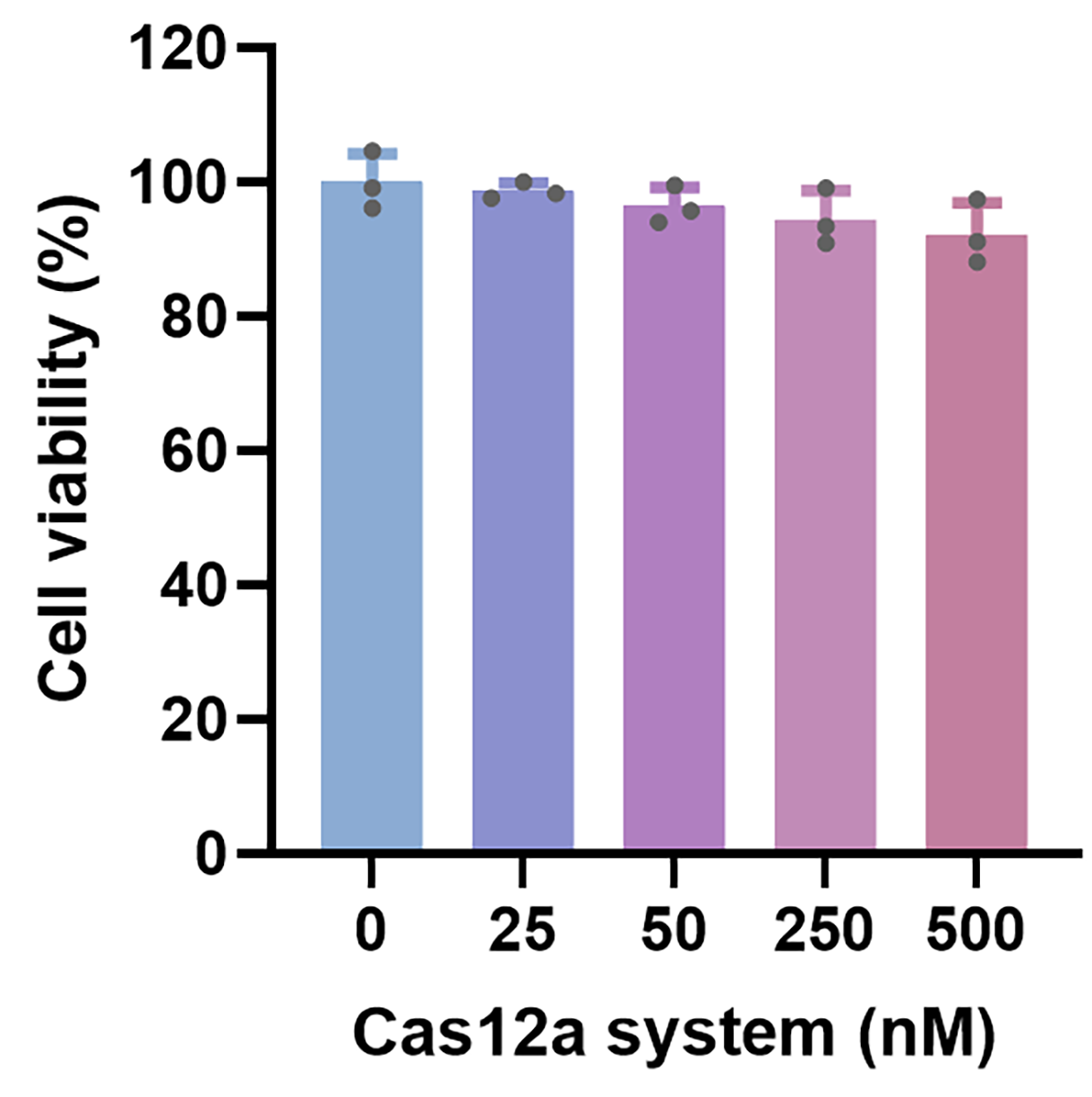


**Figure S17.** CCK-8 assay for Cas12a system cytotoxicity.


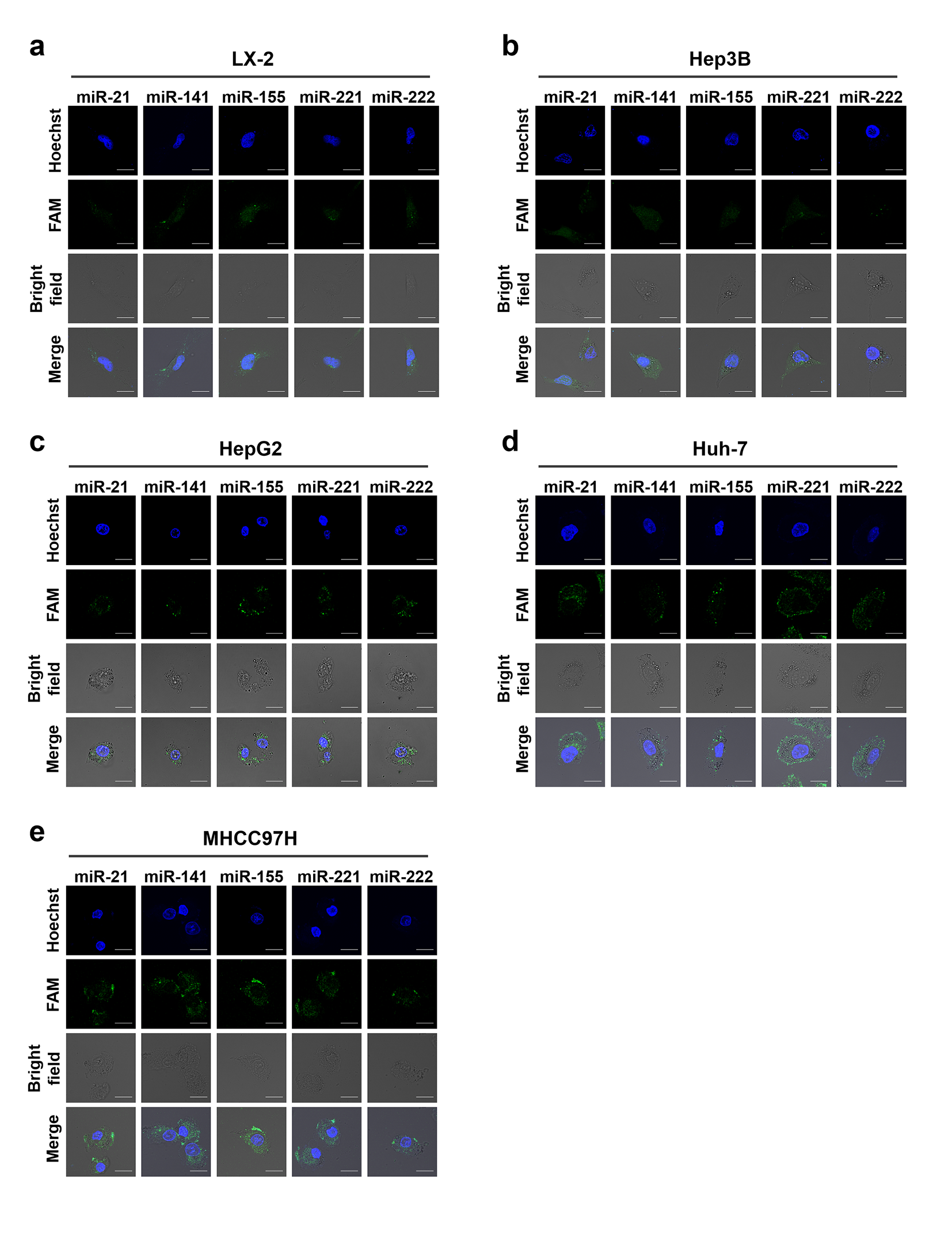


**Figure S18.** Confocal microscopic images of various miRNA in liver cell lines, including **a** LX-2, **b** Hep3B, **c** HepG2, **d** Huh-7, and **e** MHCC97H.


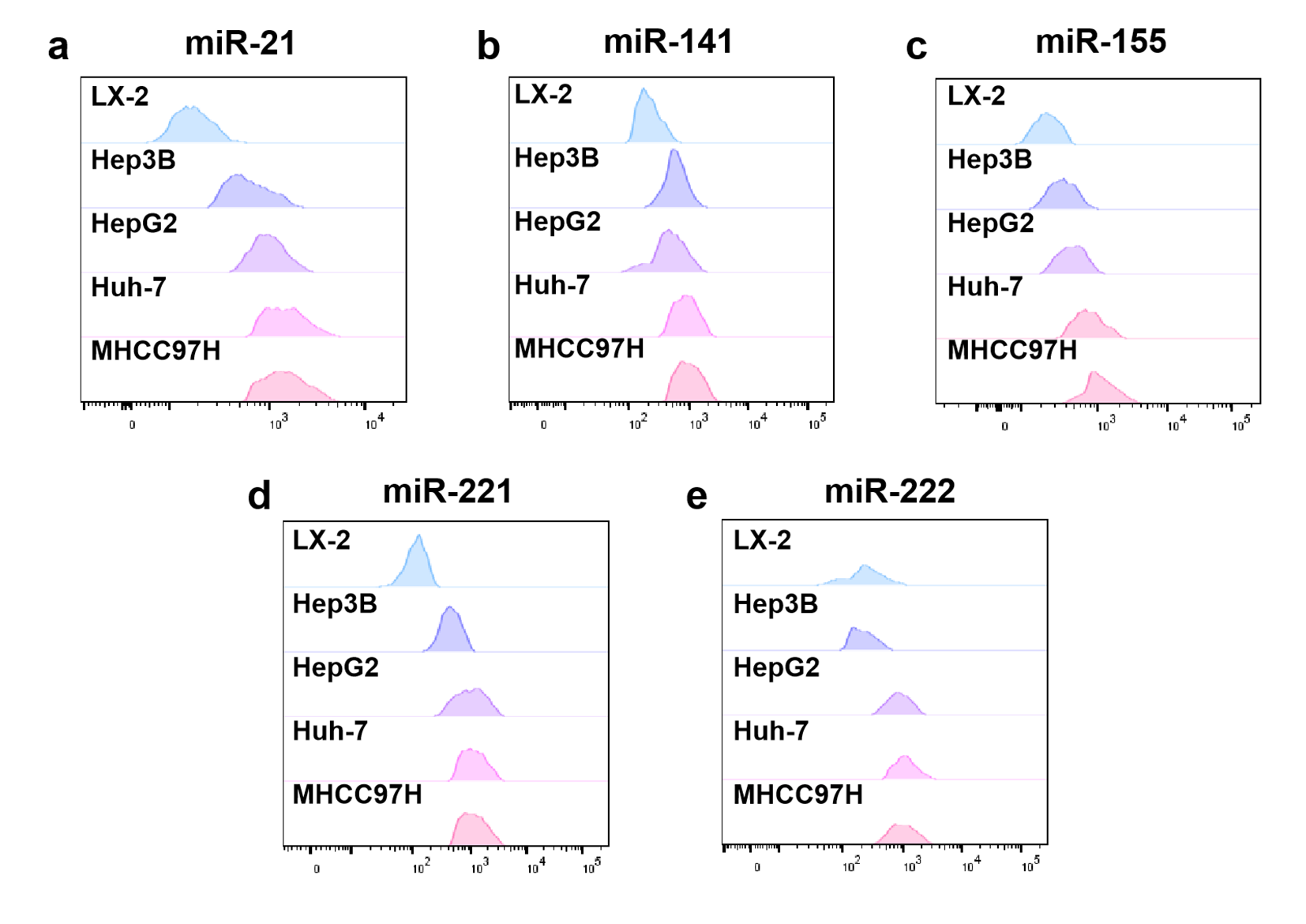


**Figure S19.** Flow-cytometry analysis of **a** miR-21, **b** miR-141, **c** miR-155, **d** miR-221 and **e** miR-222 levels in the five cell lines.


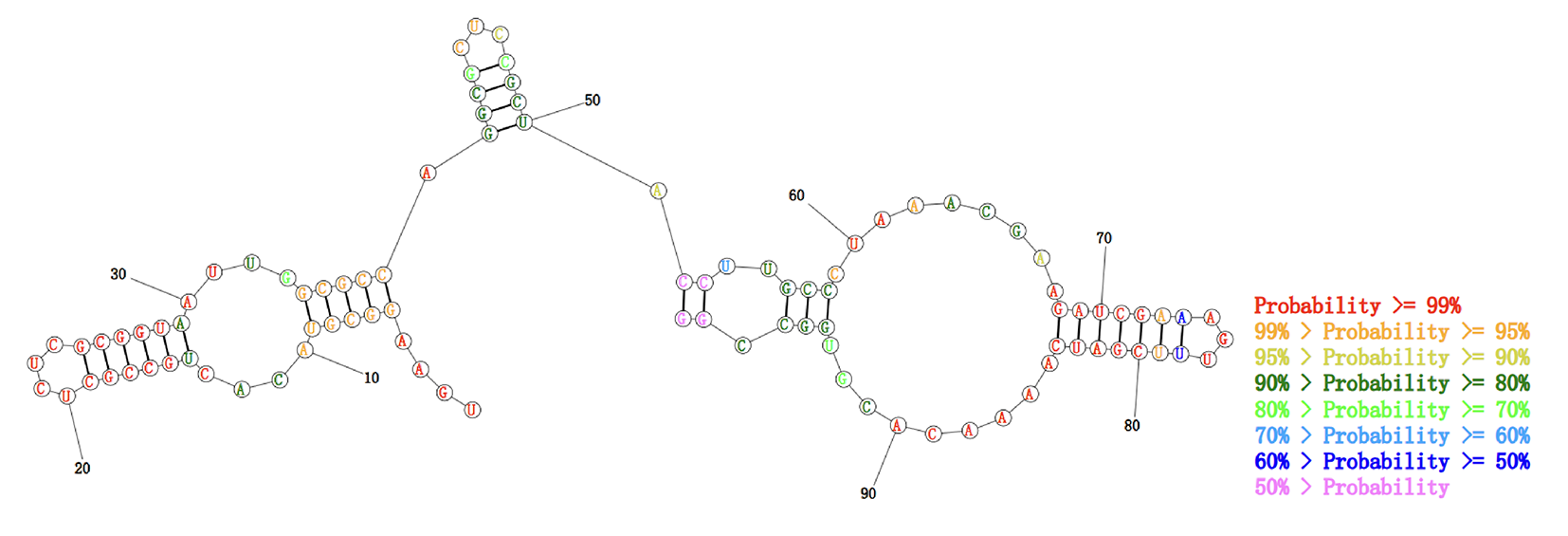


**Figure S20.** Predicted secondary structure of MS2 RNA generated by RNAfold.


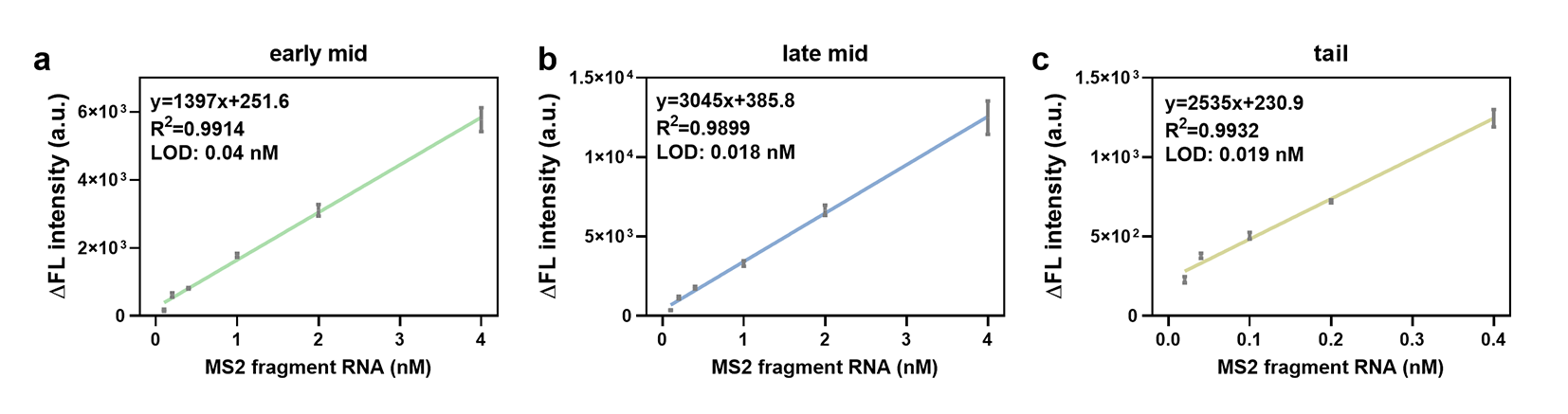


**Figure S21.** Linear response of split activator-based MS2 RNA detection at **a** early mid, **b** late mid and **c** tail sites.


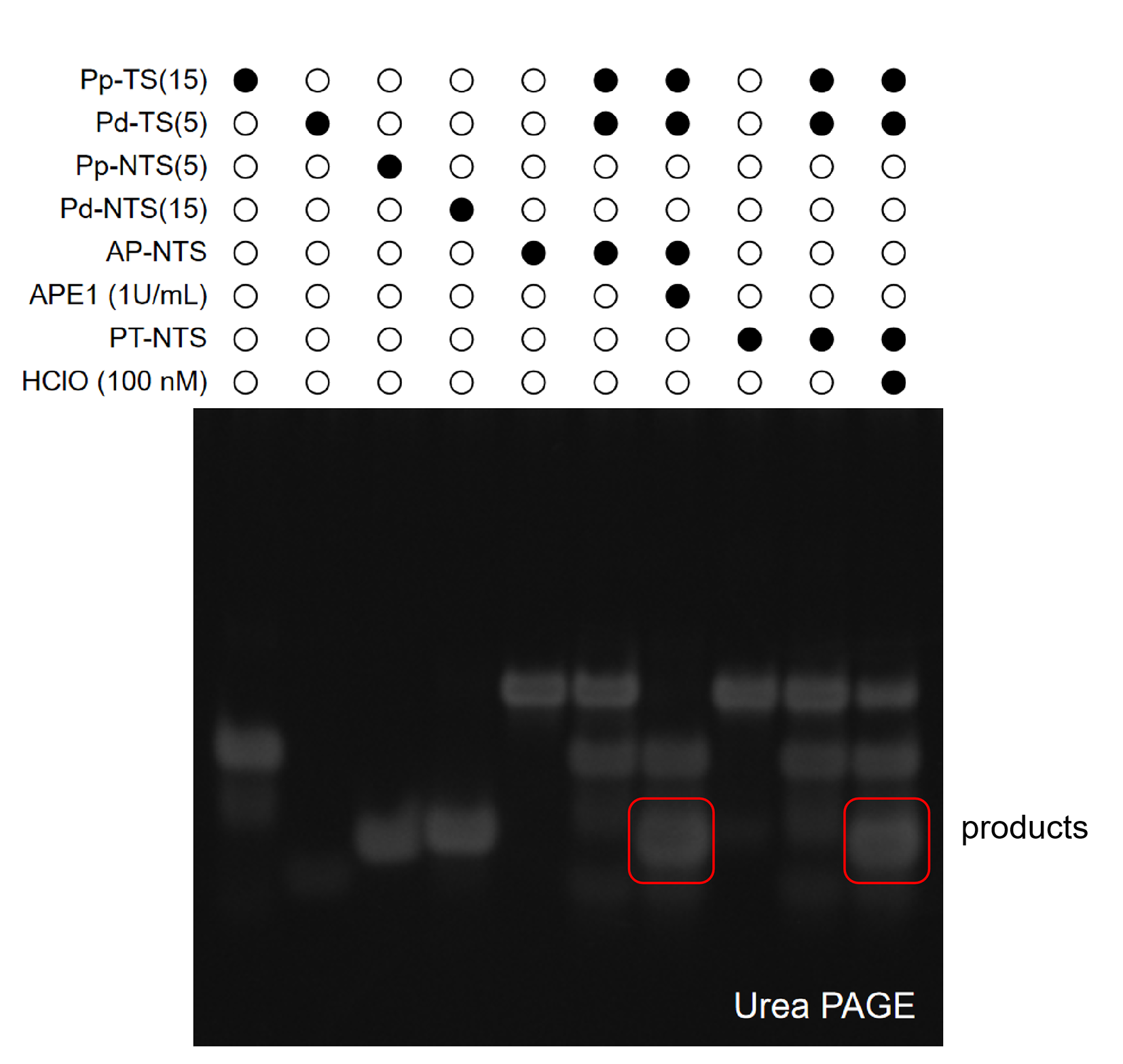


**Figure S22.** Urea-PAGE assay of APE1 and HClO cleavage products.
